# Psoriasis-Associated Inflammatory Conditions Induce IL-23 mRNA Expression in Normal Human Epidermal Keratinocytes

**DOI:** 10.3390/ijms23010540

**Published:** 2022-01-04

**Authors:** Evelyn Kelemen, Éva Ádám, Stella Márta Sági, Anikó Göblös, Lajos Kemény, Zsuzsanna Bata-Csörgő, Márta Széll, Judit Danis

**Affiliations:** 1 Department of Dermatology and Allergology, University of Szeged, 6720 Szeged, Hungary; kelemen.evelyn@med.u-szeged.hu (E.K.); sagi.stella98@gmail.com (S.M.S.); kemeny.lajos@med.u-szeged.hu (L.K.); bata.zsuzsa@med.u-szeged.hu (Z.B.-C.); 2 Department of Medical Genetics, University of Szeged, 6720 Szeged, Hungary; szell.marta@med.u-szeged.hu; 3 MTA-SZTE Dermatological Research Group, Eötvös Loránd Research Network, 6720 Szeged, Hungary; goblos.aniko@gmail.com (A.G.); danis.judit@med.u-szeged.hu (J.D.); 4 HCEMM-USZ Skin Research Group, 6720 Szeged, Hungary

**Keywords:** psoriasis, nucleic acid analogues, interleukin-23, qPCR array

## Abstract

Psoriasis is a multifactorial, chronic inflammatory skin disease, the development of which is affected by both genetic and environmental factors. Cytosolic nucleic acid fragments, recognized as pathogen- and danger-associated molecular patterns, are highly abundant in psoriatic skin. It is known that psoriatic skin exhibits increased levels of IL-23 compared to healthy skin. However, the relationship between free nucleic acid levels and IL-23 expression has not been clarified yet. To examine a molecular mechanism by which nucleic acids potentially modulate IL-23 levels, an in vitro system was developed to investigate the IL-23 mRNA expression of normal human epidermal keratinocytes under psoriasis-like circumstances. This system was established using synthetic nucleic acid analogues (poly(dA:dT) and poly(I:C)). Signaling pathways, receptor involvement and the effect of PRINS, a long non-coding RNA previously identified and characterized by our research group, were analyzed to better understand the regulation of IL-23 in keratinocytes. Our results indicate that free nucleic acids regulate epithelial IL-23 mRNA expression through the TLR3 receptor and specific signaling pathways, thereby, contributing to the development of an inflammatory milieu favorable for the appearance of psoriatic symptoms. A moderate negative correlation was confirmed between the nucleic-acid-induced IL-23 mRNA level and the rate of its decrease upon PRINS overexpression.

## 1. Introduction

Psoriasis is a multifactorial, chronic inflammatory skin disease affecting 2% of the population and has been known since ancient times. Both congenital predisposition and environmental factors play a role in its development. Raised, well-demarcated, erythematous oval plaques with silvery scales are the prominent signs of the most common form, the plaque-type psoriasis. This symptom is caused by the abnormal proliferation and differentiation of basal keratinocytes and their dysregulated interplay with professional immune cells. The plaques mostly occur on elbows, knees and the scalp, but they can also affect any other part of the body [1,2]. Plaques are surrounded by clinically healthy-looking skin, which is referred to as non-lesional or uninvolved skin. Despite the fact that the uninvolved skin of psoriasis patients appears macroscopically identical to normal skin, it contains molecular, cellular and extracellular alterations and, in several aspects, has a pre-psoriatic phenotype [3,4,5,6,7].

At different stages of the disease, a variety of innate and adaptive immune cells and proinflammatory mediators are involved. The aberrant immune and epidermal response seen in psoriasis is maintained by pathogenic crosstalk between epithelial and immune cells, and it is primarily driven by proinflammatory molecules, such as TNF-α, IL-23 and IL-17. In recent years, therapeutic targeting of these mediators has been proven to be clinically effective [8,9].

After physical trauma or infection, keratinocytes release LL-37, which is a cationic antimicrobial peptide that binds DNA and RNA fragments released by damaged skin cells. LL-37 and self-derived nucleic acids form a complex found in psoriatic lesions, and this complex activates TLR7/9-bearing plasmacytoid dendritic cells, which are normally absent from healthy skin [10,11,12]. These cells release proinflammatory cytokines (IL-1β, IL-6 and TNF) and interferons (IFNs) and through these have an important role in the development of the disease also by activating keratinocytes and myeloid dendritic cells. The number of myeloid dermal dendritic cells is elevated in psoriatic skin [13], and the mature cells migrate to skin-draining lymph nodes to present antigen to naive T-cells. T-cells are also critical for the initiation phase of the disease as their interaction with activated dermal dendritic cells is central to the development of plaque formation and the resulting creation of an IL-23/IL-17 inflammatory environment. In this environment, IL-23 derived from dendritic cells and macrophages promotes the effector functions of T helper 17 (Th17) and cytotoxic cells [1]. Taken together, these data indicate that cytosolic nucleic acid fragments, which are recognized as pathogen- and danger-associated molecular patterns (PAMPs and DAMPS), are highly abundant in the psoriatic skin and their presence can lead to the chronic activation of professional immune cells. These professional immune cells migrate into the epidermis, and their mediators stimulate excessive proliferation and abnormal differentiation of epithelial cells. This, in turn, leads to a thickening of the epidermis and the formation of inflamed plaques [14].

The role of nucleic acid fragments in psoriasis is supported by the elevated levels of cell-free DNA found in the blood of psoriasis patients, but the exact source of the nucleic acids associated with the initial inflammatory events is not yet known [15]. Reduced deoxyribonuclease activity in keratinocytes and disturbed ribonuclease activity in psoriatic skin have been observed [16,17]. Increased serum mtDNA levels originating from mitochondrial dysregulation can act as a DAMP [18,19]. In psoriatic skin, neutrophils and neutrophil extracellular traps (NETs) have also been reported as sources of nucleic acids. RNA associated with NETs might be bound by LL37, and these activate inflammatory reactions that could possibly cause a self-exciting cycle contributing to chronic inflammation in psoriasis [20]. Interferon-γ (IFN-γ), produced by Th1 and Th17 cells can also contribute to the recognition of nucleic acids. The expression of IFN-γ is elevated in psoriatic lesional skin and in the serum of the patients [21], thereby, this cytokine is able to prime nucleic-acid-induced inflammatory responses in keratinocytes and drive IL-23 expression [22].

A number of results suggested that the Th17/interleukin-23 axis plays a dominant role in the disease, as it promotes chronic inflammation. IL-23 is responsible for the development of Th17 cells, leading to a production of IL-17 and IL-22 cytokines, which are involved in the pathogenesis of psoriasis. Keratinocytes and activated antigen-presenting cells (Langerhans cells, macrophages and dendritic cells) all produce IL-23, the expression of which is elevated in psoriatic lesional skin, leading to an increased number of Th17 cells [23,24]. Recently, IL-23 targeting therapies for psoriasis have become widely used. IL-23 is a heterodimeric cytokine consisting of a unique p19 and a p40 subunit, the latter is shared with IL-12. Increased levels of these two subunits and the IL-23 receptor were found in psoriatic skin of patients, suggesting that IL-23, rather than IL-12, plays a role in disease pathogenesis [25]. Ustekinumab, a monoclonal antibody against the common p40 subunit is a highly effective treatment for psoriasis, and the antibodies targeting the p19 subunit (guselkumab, tildrakizumab and risankizumab) also show promising impact for symptom amelioration [1,26].

Although the literature reports high levels of IL-23 and free nucleic acids in psoriatic skin, it is not known yet whether there is an association between these participants in the development of the disease or whether they act independently.

To address this question, an in vitro system was developed to investigate IL-23 production of normal human epidermal keratinocytes (NHEKs) under psoriasis-like circumstances established with the introduction of the synthetic nucleic acid analogues poly(dA:dT) and poly(I:C). Signaling pathways, receptor involvement and the effect of the long non-coding RNA PRINS were analyzed to examine the regulation of this cytokine in keratinocytes.

At the beginning of the 2000s, our research group identified a long non-coding RNA, referred to as psoriasis-susceptibility-related RNA gene induced by stress (PRINS), which exhibited higher levels in the uninvolved skin of psoriatic patients than in lesional or healthy epidermis. PRINS has a protective role in cells exposed to stress [5]. It has also been shown that PRINS binds directly to the mRNAs of IL-6 and CCL-5 (RANTES) at specific binding sites and eventually destabilizes these mRNAs, leading to a decrease in their accumulation. Based on this, we conclude that PRINS has a restrictive role in inflammatory processes [5,14,27]. Our preliminary in silico data also suggested that PRINS contains an IL-23 mRNA binding site. However, there are currently no data in the literature about the possible connection between the high IL-23 level in psoriatic skin and the protective role of PRINS in inflammatory conditions.

Our results indicate that free nucleic acids regulate epithelial IL-23 production through the TLR3 receptor and specific signaling pathways, thereby, contributing to the development of the inflammatory milieu favorable for the appearance of psoriatic symptoms. Additionally, we demonstrated that PRINS, which was previously identified in the uninvolved epidermis of psoriatic patients as a protective factor, contributes to these processes with a high interindividual variability.

## 2. Results

### 2.1. Free Nucleic Acid Analogues Induce Increased IL-23 mRNA Expression in Human Keratinocytes

#### 2.1.1. Psoriasis-Relevant Gene Expression Pattern Is Modulated by Free Nucleic Acid Analogues

**Figure 1:**
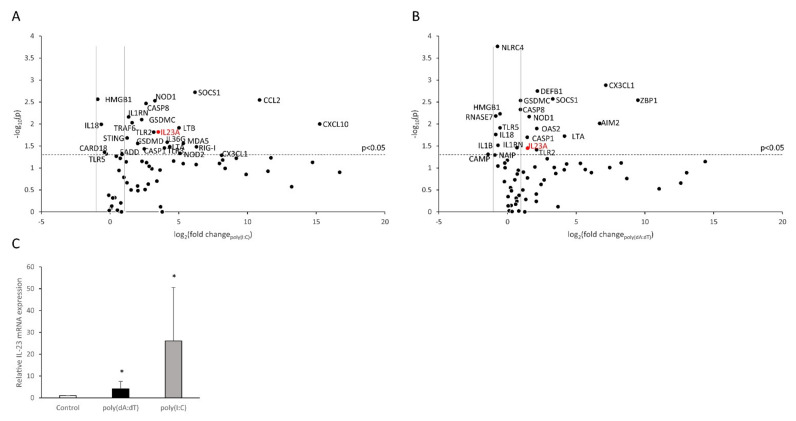
Changes in keratinocyte gene expression induced by poly(I:C) (**A**) and poly(dA:dT) (**B**). RT-PCR validation on independent samples revealed that NHEKs respond to poly(I:C) and poly(dA:dT) with increased IL-23 mRNA expression (**C**). To assess multiple gene expression changes upon inflammatory stimuli, a qPCR array of inflammatory mediators and receptors was carried out on normal human epidermal keratinocyte samples transfected by 0.666 μg/mL poly(I:C) (**A**) or 1 μg/mL poly(dA:dT) for 12 h (**B**) and expression changes were compared to mock-transfected samples. Results are presented as volcano plots, significant changes (*p* < 0.05) are displayed above the horizontal dashed line. Vertical lines indicate a ±2-fold change in expression compared to mocktransfected control samples. Four independent experiments were carried out, statistical significance was calculated by Student’s *t*-test with correction for multiple comparisons (**A**,**B**). For validation, NHEKs were transfected with 0.666 μg/mL poly(I:C) and 1 μg/mL poly(dA:dT), samples were collected 12 h after treatments. Relative IL-23 mRNA expression was determined by the ∆∆Ct method, normalized to GAPDH mRNA expression and compared to the expression of the time-matched mock-treated (Control) samples. Data are presented as mean of six independent experiments ± SD. Significance was determined by one-tailed, paired Student’s-test with correction, * *p* < 0.05 compared to control (**C**).

To identify genes responding with altered expression in NHEKs to treatment with the synthetic nucleic acid analogues poly(I:C) and poly(dA:dT), a qPCR array containing 84 psoriasis-relevant genes (see list of genes as Supplementary Table S1), including cytokines, chemokines, antimicrobial peptides and cytoplasmic receptors, was performed. In this experiment, we observed that 14 genes were not expressed by the cells, whereas the expression of 15 additional genes, mostly antimicrobial peptides, was not altered after treatment with either poly(I:C) or poly(dA:dT). We identified 37 genes that were induced by both treatments, including cytokines, chemokines and pattern recognition receptors (PRRs). Increased expression only in response to poly(I:C) treatment was observed for seven additional genes (Figure 1A,B). 

Most importantly, we observed that, in a manner similar to other inflammatory cytokines, such as IL-6 and TNF-α (results previously published in [28], and Supplementary Figure S1B) IL-23 expression increased in response to both poly(dA:dT) and poly(I:C) treatment. Since IL-23 is known to play an important role in psoriasis pathogenesis, we designed further experiments to examine the molecular mechanism of free nucleic acid–induced IL-23 transcription in human keratinocytes.

In the qPCR array, mRNA samples derived from four healthy donors were used, and the first validation experiments were carried out on the same mRNA set. These results confirmed that poly(I:C) induced significant increase in IL-23 mRNA, whereas poly(dA:dT) induced an upward trend in expression (Supplementary Figure S1A,B). To expand the validation of the array, we included additional independent donors (*n* = 6) and observed significantly higher IL-23 expression after both treatments; however, the treatment with poly(I:C) had a more pronounced effect in this experiment as well (Figure 1C).

#### 2.1.2. Poly(I:C) and Poly(dA:dT) Induce IL-23 mRNA Expression with Different Kinetics

**Figure 2:**
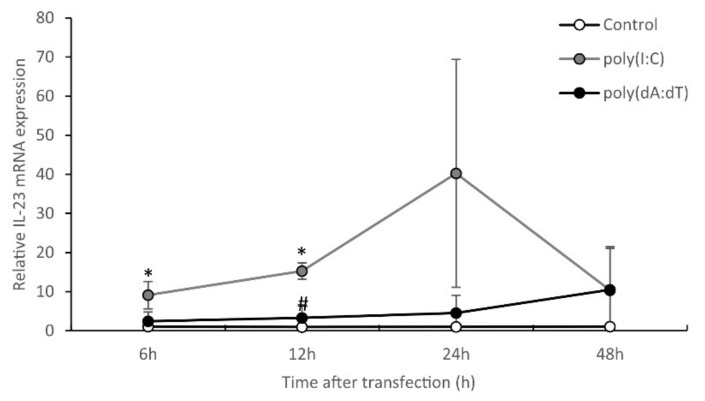
Kinetics of the IL-23 mRNA expression in NHEKs upon nucleic acid induction. Cells were transfected with 0.666 μg/mL poly(I:C) and 1 μg/mL poly(dA:dT), and samples were collected at 6, 12, 24 and 48 h after transfection. Relative IL-23 mRNA expression was determined by the ∆∆Ct method, normalized to GAPDH mRNA expression and compared to the expression of the untreated (Control) 0 h samples. Data are presented as means of three independent experiments ± SD. Significance was determined by one-tailed, paired Student’s *t*-test, * *p* < 0.05 poly(I:C)-treated vs. time-matched control samples, # *p* < 0.05 poly(dA:dT)-treated vs. time-matched control samples.

Previously, we identified kinetic differences in poly(I:C)- and poly(dA:dT)-induced inflammatory responses in keratinocytes [28]. To clarify this in the case of IL-23, we measured IL-23 mRNA levels at different time points after nucleic acid treatments. Compared to the previously investigated inflammatory mediators, such as IL-6 or TNF-α, which peak at 6 to 12 h after poly(I:C) or (dA:dT) treatment [28] (Supplementary Figure S2), peak expression of IL-23 was observed later, at 24 h after poly(I:C) transfection, while a slowly rising tendency was detected after poly(dA:dT) treatment and the level was lower than that observed after poly(I:C) treatment (Figure 2).

#### 2.1.3. Psoriasis-Specific Stimuli Induce IL-23 mRNA Expression

**Figure 3:**
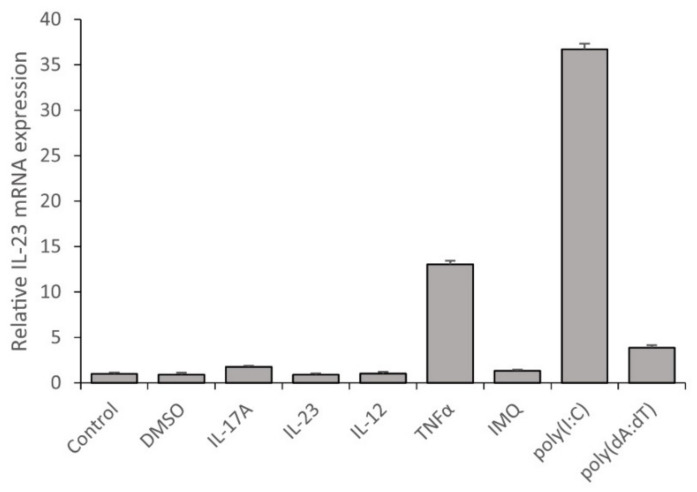
Synthetic nucleic acid analogues and TNFα induce IL-23 mRNA expression in NHEKs. Cells were transfected with 0.666 μg/mL poly(I:C), 1 μg/mL poly(dA:dT), 2 μL DMSO, 1 μg/mL IL-17A, 1 μg/mL IL-23, 1 μg/mL IL-12, 0.5 μg/mL TNFα and 1.33 μg/mL IMQ. Relative IL-23 mRNA expression was determined by the ∆∆Ct method, normalized to GAPDH mRNA expression and compared to the expression of the untreated control samples. Results of one representative experiment are shown, data are presented as the average of three technical repetitions ± SD.

In psoriatic skin, keratinocytes are exposed not only to free nucleic acids but to numerous cytokines, which shape the inflammatory response of the keratinocytes. To compare the effects of psoriasis-specific-cytokines with the effects of nucleic acids on IL-23 mRNA expression in keratinocytes, we used psoriasis-relevant cytokines, such as IL-17A, IL-12, TNF-α and IL-23, and synthetic nucleic acid analogues imiquimod (IMQ), poly(dA:dT) and poly(I:C), since these molecules have long been known to have a role in psoriasis pathogenesis. The highest IL-23 expression was induced by poly(I:C), whereas poly(dA:dT) and TNF-α treatment also elevated IL-23 transcription but to a lesser extent (Figure 3). Other psoriasis-relevant cytokines and IMQ, which is used to induce psoriasis in mouse models [29], did not affect IL-23 mRNA expression in keratinocytes.

Interestingly, although IL-23 is thought to originate from professional immune cells [30], in our experiments THP-1 macrophages and Jurkat T-cells failed to exceed the level of IL-23 mRNA production of keratinocytes in response to poly(I:C), poly(dA:dT) and TNFα (data not shown). These results indicate that synthetic nucleic acids modeling free cellular nucleic acids—especially poly(I:C)—play an important role in the psoriasis-associated inflammatory processes by inducing elevated IL-23 levels in human keratinocytes.

### 2.2. Free Nucleic Acids Act through Specific Receptors and Signaling Pathways to Modulate IL-23 mRNA Expression Levels

#### 2.2.1. TLR3 Is the Main Nucleic Acid Sensing Receptor Conveying IL-23 mRNA Expression

**Figure 4:**
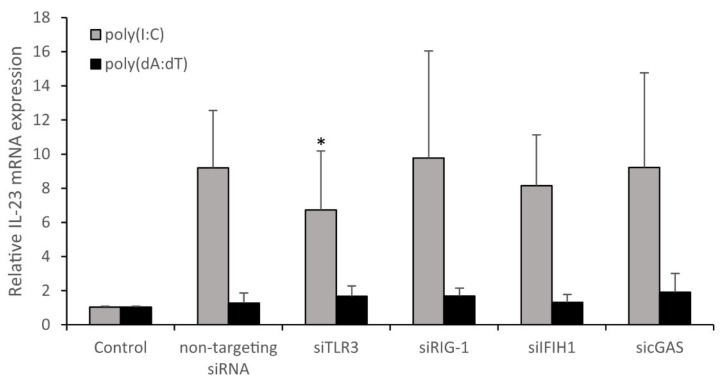
Silencing of the TLR3 pattern recognition receptor affects poly(I:C)-induced IL-23 mRNA expression of NHEKs. Expression of pattern recognition receptors were silenced with siRNA-mediated inhibiton for 24 h, and subsequently, the cells were transfected with 0.666 μg/mL poly(I:C) and 1 μg/mL poly(dA:dT). Relative IL-23 mRNA expression was determined by the ∆∆Ct method, normalized to GAPDH mRNA expression and compared to the expression of the non-treated control samples. Data are presented as means of four independent experiments ± SD. Significance was determined between siRNA-transfected samples and the non-targeting siRNA-transfected samples by the one-tailed, paired Student’s *t*-test with correction for multiple comparisons * *p* < 0.05.

In psoriasis, nucleic acid fragments originating from tissue or pathogens are present and recognized as pathogenic factors for disease development [15,17,19]. Several PRRs that recognize these RNA and DNA fragments and induce inflammatory mechanisms have already been identified [31,32,33], and many of these are expressed in both keratinocytes and professional immune cells. We aimed to examine which of these receptors plays an important role in mediating nucleic-acid-induced IL-23 mRNA expression in keratinocytes. To this end, siRNA-mediated silencing was carried out to decrease the level of TLR-3, RIG-I, IFIH1(MDA-5) and cyclic GMP-AMP synthase (cGAS) mRNA (Supplementary Figure S3). Our results showed that poly(I:C)-induced IL-23 mRNA expression is mediated primarily by TLR3. The silencing of the other receptors in our experiments did not affect the influence of poly(dA:dT) on IL-23 transcription (Figure 4 and Supplementary Figure S4A–D).

#### 2.2.2. Multiple Pathways Transmit Free Nucleic Acid Signals to Mediate IL-23 mRNA Expression

**Figure 5:**
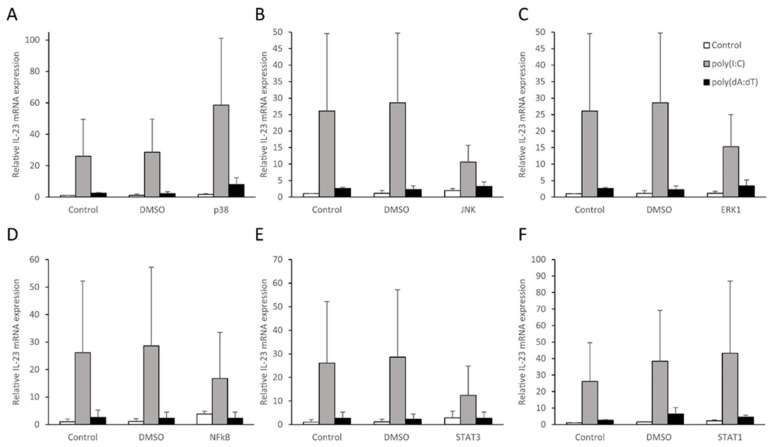
Inhibition of the JNK, ERK1, NF-κB and STAT3 pathway components had an effect on the nucleic-acid-induced IL-23 mRNA expression of NHEK cells (**A**–**F**). Specific inhibiton of signaling pathway components was used for 1 h with the cells being subsequently transfected with 0.666 μg/mL poly(I:C) and 1 μg/mL poly(dA:dT) for 24 h. Equal concentration of DMSO was used as solvent control for each inhibitor, demonstrating no difference from non-pretreated control samples. Relative expression was determined by the ∆∆Ct method, normalized to GAPDH mRNA expression and compared to the expression of the untreated control samples. Data are presented as mean of three independent experiments ± SD. Significance was tested by one-tailed, paired *T*-test comparing to DMSO-treated solvent-control samples. In spite of obvious differencies in the values, no significance could be demonstrated among them due to high standard deviations of the independent experiments performed on NHEKs derived from independent volunteers.

Our next aim was to identify the downstream signaling pathways through which elevated nucleic acid levels lead to increased IL-23 mRNA synthesis in keratinocytes. In our studies, we examined six pathways that were previously shown to be affected by nucleic acids [34]. The activity of a major component of each of these pathways was decreased by applying specific inhibitors, such as Bay 11-7085 for NF-κB, PD95089 for dual-specificity mitogen-activated protein kinase kinase1 and 2, SB203580 for p38, SP600125 for JNK, fludarabine for STAT-1 and Stattic for STAT-3. Keratinocytes were preincubated with the inhibitor for an hour before transfection with poly(I:C) or poly(dA:dT). Our results showed that poly(I:C)-induced IL-23 mRNA expression is mediated by JNK, ERK1/2, NF-κB and STAT3 pathways, downstream of TLR3 receptor (Figure 5 and Supplementary Figure S5A–C). However, the inhibition of either of these pathways had no effect on poly(dA:dT)-induced IL-23 expression, which might also be due to the low IL-23 induction triggered by poly(dA:dT).

### 2.3. The Effect of the PRINS Long Non-Coding RNA Shows a Great Interindividual Difference on Nucleic Acid Analogue–Induced IL-23 mRNA Expression in Human Keratinocytes

**Figure 6:**
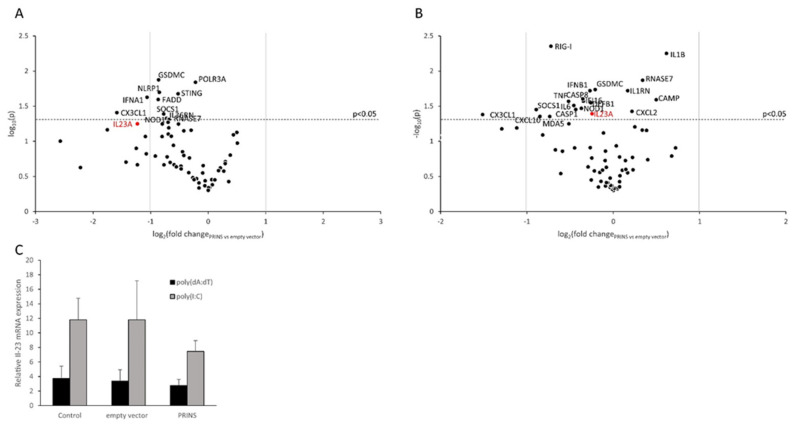
Gene expression changes upon the overexpression of the PRINS long non-coding RNA in NHEKs transfected with poly(I:C) (**A**) and poly(dA:dT) (**B**). RT-PCR validation of the array results regarding nucleic-acid-induced IL-23 mRNA expression upon PRINS overexpression in NHEKs (**C**). To assess multiple gene expression changes, a qPCR array of inflammatory mediators and receptors was carried out on NHEK cells transfected by a pcDNA3.1(+) vector containing the PRINS sequence or the empty verctor. Subsequently, 24 h later, PRINS or emtpy-vector-carrying cells were transfected by 0.666 μg/mL poly(I:C) (**A**) or 1 μg/mL poly(dA:dT) (**B**) for 12 h. Expression changes in PRINS-overexpressing samples were compared to expression values of empty-vector-transfected samples. Results are presented as volcano plots, significant changes (*p* < 0.05) are displayed above the horizontal dashed line. Vertical lines indicate a ±2-fold change in expression compared to empty-vector-transfected cells. Experiments were carried out on four independent samples derived from healthy voluteers, statistical significance was calculated by Student’s *t*-test with correction for multiple comparisons (A and B). Cells overexpressing PRINS were transfected with 0.666 μg/mL poly(I:C) and 1 μg/mL poly(dA:dT). Relative IL-23 mRNA expression was determined by the ∆∆Ct method, normalized to GAPDH mRNA expression and compared to the expression of the untreated (Control) samples. For the RT-PCR experiment, we used the same total mRNA sample set (*n* = 4) as for the array experiment. Data are presented as mean of four independent experiments ± SD. Statistical significance was not found as assesed by one-tailed, paired Student’s *t*-test (**C**).

Connection between PRINS and psoriasis has been described in several earlier studies [5,7,27,35]; for example, through the regulation of certain inflammatory mediators (IL-6 and CCL5) by PRINS in nucleic-acid-induced inflammatory reactions. To identify additional targets for PRINS-mediated regulation in NHEKs, a qPCR array was used to identify genes showing altered expression as a consequence of nucleic acid challenge and PRINS overexpression (see list of genes included in the qPCR-array as Supplementary Table S1). A PRINS overexpressing construct was transiently transfected into NHEK cells, and the cells were subsequently treated with poly(I:C) or poly(dA:dT) to identify how PRINS overexpression alters the nucleic-acid-induced gene expression in keratinocytes. In the qPCR-array, impaired upregulation of inflammatory genes, including cytokines, chemokines, receptors and effector molecules, was observed. Among others, NLRP1, GSDMC, CX3CL1 and SOCS1 exhibited the most prominent changes in expression (Figure 6A,B). We have previously shown that PRINS binds to the mRNA of IL-6 and CCL-5, leading to their degradation [14]. These mRNAs were also downregulated in the qPCR array, validating our results. The qPCR array identified another psoriasis-related transcript, IL-23 mRNA, that was downregulated in the PRINS-overexpressing keratinocytes. We confirmed this result by validating the array data with keratinocytes from the same donors as used for the qPCR array (Figure 6C).

**Figure 7:**
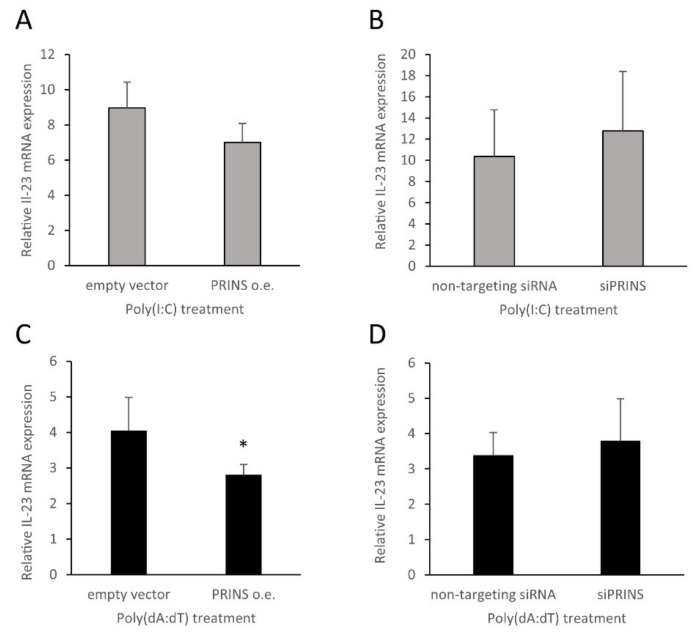
The effect of PRINS overexpression (**A**,**C**) and silencing (**B**,**D**) on the IL-23 mRNA expression upon poly(I:C) (**A**,**B**) and poly(dA:dT) (**C**,**D**) treatment. PRINS-overexpressing (**A**,**C**) or -silenced (**B**,**D**) cells were transfected with 0.666 μg/mL poly(I:C) or 1 μg/mL poly(dA:dT) Relative IL-23 mRNA levels were determined by the ∆∆Ct method, normalized to GAPDH mRNA expression and compared to mock-transfected samples. Data are presented as means of eight (**A**), fifteen (**C**) and three (**B**,**D**) independent experiments ± SD. Statistical significance was assessed by one-tailed, paired Student’s *t*-test, * *p* < 0.05.

**Figure 8:**
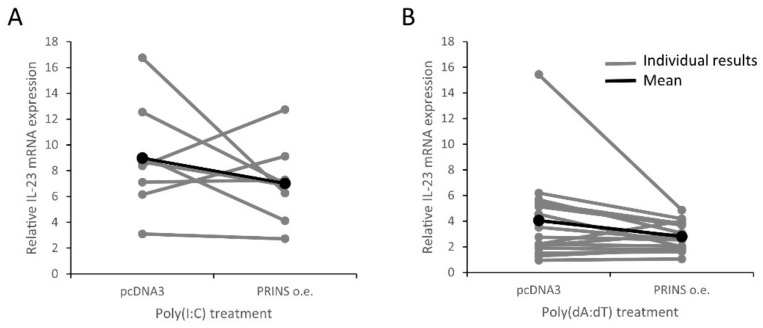
The effect of PRINS overexpression on poly(I:C)- (**A**) and poly(dA:dT)- (**B**) induced IL-23 mRNA expression in NHEKs isolated from the epidermis of 8 (**A**) and 15 (**B**) healthy volunteers. PRINS overexpressing cells were transfected with 0.666 μg/mL poly(I:C) (**A**), *n* = 8, or with 1 μg/mL poly(dA:dT) (**B**), *n* = 15. Relative IL-23 mRNA expression was determined by the ∆∆Ct method, normalized to GAPDH mRNA expression and compared to mock-transfected samples. Expression values of individual samples are shown in gray, the mean values of the individual results is shown in black.

To further support the role of PRINS in IL-23 mRNA regulation, we examined the effect of the silencing or overexpression of PRINS on the nucleic-acid-induced IL-23 mRNA expression in NHEK cells derived from additional individuals. These experiments confirmed that PRINS overexpression significantly reduced IL-23 mRNA expression after poly(dA:dT) treatment, whereas a decreasing trend was observed after poly(I:C) treatment (Figure 7A,C). We observed an opposite tendency when PRINS expression was decreased by silencing (Figure 7B,D and Supplementary Figure S6A,B), which suggests that PRINS might have a role in the nucleic-acid-induced IL-23 expression of keratinocytes. However, statistical significance was not reached in many cases, since large interindividual differences between the samples derived from independent donors can be observed (Figure 8).

Bioinformatic analysis of IL-6 and CCL-5 predicted an mRNA–lncRNA binding site [14]. Therefore, we attempted to determine whether a PRINS interaction site is present in IL-23 mRNA. The predicted binding site in PRINS to the IL-23 mRNA partly overlaps the previously identified IL-6 mRNA binding sequence (Supplementary Figure S4). Since IL-23 is an extremely polymorphic gene, we hypothesized that a reason for the large interindividual differences observed between the donors might be due to single-nucleotide variants (SNPs) at the putative PRINS interaction site in IL-23, which could interfere with PRINS binding. Moreover, SNPs of the *IL-23* gene are linked to psoriasis susceptibility [36,37,38]. Searching the NCBI (National Center for Biotechnology Information) database for polymorphisms at the putative binding site, 13 SNPs were identified on the *IL-23* gene (https://www.ncbi.nlm.nih.gov/nuccore/NM_016584.3 accessed on 1 September 2018). We sequenced the putative binding site of the *IL-23* gene from each donor and compared the sequences. Sequence variants did not correlate with the IL-23 response to PRINS overexpression. Subsequently, all exons of the *IL-23* gene were sequenced for the enrolled donors, and again no association between haplotype and the differential effect of PRINS on IL-23 mRNA abundance was observed (data not shown).

**Figure 9:**
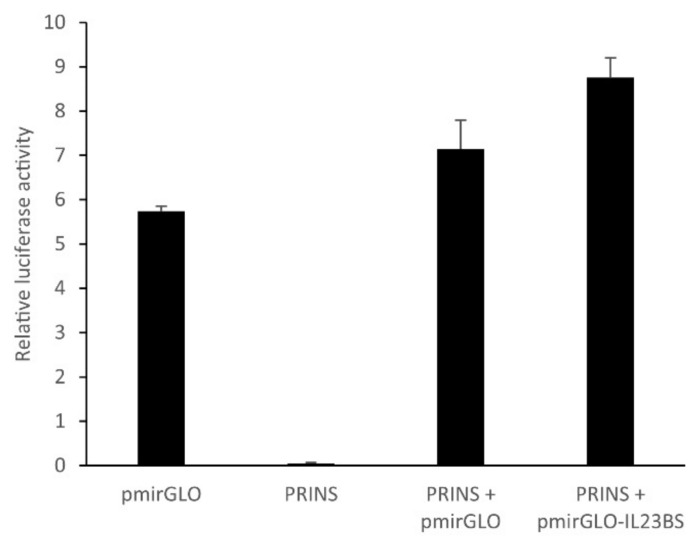
An in vitro binding assay revealed no interaction between the putative PRINS-interacting sequence on IL-23 and PRINS long non-coding RNA. Cells were transfected with PRINS pcDNA3.1 and pmirGLO plasmids as controls or co-transfected with the PRINS pcDNA3.1 plasmid in combination with pmirGLO or pmirGLO-IL23BS vectors. The pGL4.75 [hRluc/CMV] plasmid (Promega) was used as internal control. Cells were harvested after 24 h, and luciferase activities in the lysates were measured using the Firefly and Renilla Dual Luciferase Assay Kit and SYNERGY/HTX Multi-Mode reader following the manufacturer’s instructions. The luciferase activity derived from the pmirGLO plasmid was normalized to the renilla luciferase activity. Results of a representative experiment are shown with three technical repetitions.

To confirm the putative binding between PRINS and the IL-23 mRNA identified in silico, we used a luciferase-based vector carrying cloning sites at the 3′ end of the luciferase gene. The putative PRINS-interacting sequence of the IL-23 cDNA (Supplementary Figure S7) was inserted into this vector (pmirGLO-IL23BS). In the absence of any binding partner, transfected cells exhibit luciferase activity. If a binding event were to occur between PRINS and the IL-23 mRNA sequence, the luciferase mRNA would be destabilized, and luciferase activity would not be detected. Upon co-transfection of the cells with pmirGLO-IL23BS- and PRINS-expressing plasmids, we observed hardly any difference in luciferase activity in the presence and absence of the interaction site, indicating no physical interaction through this sequence between IL-23 mRNA and PRINS (Figure 9).

**Figure 10:**
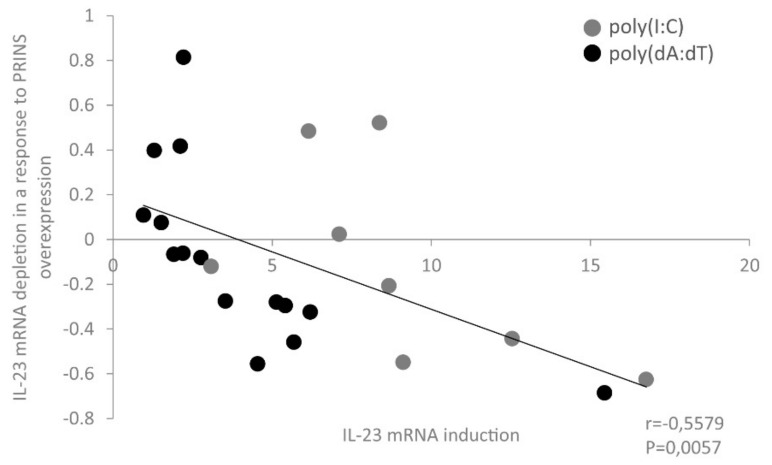
Analysis of the association between nucleic-acid-induced IL-23 mRNA abundance and the effect of PRINS overexpression on it shows a significant moderate negative correlation. The IL-23 mRNA depletion was determined as the relative change of PRINS-overexpressing samples compared to pcDNA3.1-transfected samples and displayed as a function of the poly(I:C)- or poly(dA:dT)-induced IL-23 mRNA expression of each sample.

However, we noticed that IL-23 mRNA downregulation by PRINS overexpression was more apparent in those donor samples where initial induction of IL-23 by nucleic acids was the highest (Figure 8), suggesting a correlation. Pearson’s correlation analysis confirmed a significant, moderate, negative correlation between the extent of IL-23 mRNA expression by nucleic acid induction and the rate of decrease upon PRINS overexpression (Figure 10). This suggests that an adequately high level of IL-23 mRNA is required for the regulatory effect of PRINS on IL-23 mRNA expression.

## 3. Discussion

Nucleic acid fragments are important PAMPs or DAMPs that induce the innate immune processes of professional and non-professional immune cells [34,39]. Activation of inflammasomes in keratinocyte dendritic cells mediates the promotion of inflammation [10], and the accumulation of RNA and DNA fragments in keratinocytes has a pathogenic role in the psoriatic parakeratosis [40,41]. Several studies suggested that the Th17/IL-23 axis plays a role in disease pathogenesis [29,42,43]. Increased expression of the subunits of IL-23 and its receptor were found in psoriatic lesional skin, which suggest that this cytokine—and not IL-12—play a role in psoriatic inflammation [26,44,45]. However, there are no available data about the possible connection between the effect of these inflammatory agents. 

In this study, we aimed to investigate the mechanism of how nucleic acid challenge regulates IL-23 mRNA expression in keratinocytes. Our results showed that both poly(I:C) and poly(dA:dT) induce IL-23 mRNA expression in keratinocytes but to a different degree and with different kinetics. The difference might originate from differences in th e recognition of RNA and DNA by PRRs. Specific PRRs that are capable of sensing nucleic acids in keratinocytes have already been widely described. These receptors recognize RNA and DNA fragments of both native and pathogenic origin and induce inflammatory mechanisms. TLR3 recognizes dsRNAs from viruses as well as poly(I:C), and it is essential for the production of the IL-12p40 subunit [31]. TLR3 was also shown to induce IL-23p19 expression through interferon regulatory factor 6 (IRF6) [22]. RIG-I and IFIH1 belong to the RIG-like receptors [31]. RIG-I predominantly recognizes short dsRNA, while IFIH1 senses long dsRNA from viruses. However, it was previously shown that poly(dA:dT) is transcribed into dsRNA by RNA polymerase III before recognition by the RIG-I receptor [28,46], which, as suggested previously, might explain the kinetic differences between poly(I:C) and poly(dA:dT) treatment [28]. Recently, cGAS was described as the major PRR to recognize cytosolic DNA fragments [32,33]. To date, the involvement of these receptors in the production of IL-23 by keratinocytes has not been addressed. Our results demonstrated that, whereas poly(I:C)-mediated IL-23 mRNA expression was decreased by silencing TLR3, in line with previous reports [22], silencing of none of the studied receptors had an effect on poly(dA:dT)-induced IL-23 mRNA expression. Nevertheless, we emphasize that the level of induction after poly(dA:dT) treatment never approached the level induced by poly(I:C) treatment, which might explain the negative results in the silencing experiments. Our experiments were carried out on NHEK cells isolated from four healthy donors, and average values are depicted in Figure 4. However, even in this small dataset, we observed differences in the reactions of the individuals, especially in the case of donors NHK49 and 51, in which IFIH1 silencing resulted in a remarkable decrease in the IL-23 mRNA level (Supplementary Figure S2A–D). These results drew our attention to the importance of the differential sensitivity of individuals in responses to different agents.

The nuclear-factor (NF)-κB signaling pathway affects cell survival, proliferation and anti-apoptotic effects of lymphocytes and keratinocytes, and it is known that TNF-α induces Th17 to produce pro-inflammatory cytokines through this pathway in psoriatic lesions [23]. In eukaryotic cells, three mitogen-activated protein kinase (MAPK) cascades have been identified: ERK, JNK and p38. The ERK signaling pathway plays an important role in cell proliferation and differentiation, while JNK and p38 are mainly related to stress and apoptosis of cells [47]. The JAK/STAT signaling pathway, also known as the IL-6 signaling pathway, is involved in many biological processes, such as cell proliferation, differentiation and apoptosis, and is also closely related to many immune and inflammatory diseases [48]. It has been shown that, in human keratinocytes, poly(I:C) induces NF-κB, p38 and STAT-1 signaling [34], whereas poly(dA:dT) treatment activated NF-κB, p38 and JNK signaling in human melanocytes [49]. Recent studies show that increased levels of oxidative products activate keratinocytes, Th1 and Th17 cells through the MAPK, NF-kB and JAK-STAT pathways, resulting in the production of several cytokines which are involved in psoriatic inflammation [50,51,52].

Inhibition of the ERK-1, JNK, NF-κB and STAT3 pathways resulted in a decrease in poly(I:C)-induced IL-23 mRNA expression of keratinocytes, suggesting that all of these pathway components play a role in the RNA-induced production of IL-23 mRNA under inflammatory circumstances. Blockage of these pathways did not result in complete decay of IL-23 expression, showing the involvement of other, related pathways including the known role of TLR3 signaling through IRF6 [22]. These suggests that poly(I:C)-induced IL-23 expression in keratinocytes is mainly mediated by TLR3-driven activation of several parallel pathways. Interestingly, similarly to the silencing of the major nucleic-acid-sensing PRRs, inhibition of these major pathways did not have an effect on poly(dA:dT)-induced IL-23 mRNA expression, suggesting the importance of alternative receptors and pathways in DNA-induced IL-23 mRNA expression that have not yet been identified. The involvement of other, less exposed receptors or pathways in poly(dA:dT)-induced IL-23 mRNA expression would also explain the observed lower expression compared to poly(I:C)-induced expression levels.

PRINS long non-coding RNA has been characterized as a contributor to the anti-inflammatory state of the epidermis by binding directly to inflammatory molecules, specifically to IL-6 and CCL-5 mRNAs [14,35]. To identify additional target transcripts of PRINS under inflammatory conditions, nucleic acid induction was applied during PRINS overexpression. PRINS overexpression led to a decrease in nucleic-acid-induced IL-23 mRNA expression, and a putative lncRNA–mRNA binding site was identified in silico. Although initially we confirmed the decrease in IL-23 mRNA expression upon PRINS overexpression, increasing the number of samples led us to conclude that there were large interindividual differences among the independent donors. Since a direct lncRNA–mRNA interaction could be affected by SNPs, their presence was assessed; however, sequencing the putative binding site of the different donors revealed no correlation with the presence of sequence variants and the IL-23 mRNA response to PRINS overexpression. Interaction could not be confirmed with an experimental approach to evaluate the direct interaction between the IL-23 mRNA putative binding site and the PRINS lncRNA molecule using a luciferase-based binding assay. In further studies, the responses of the donors seemed to be related to the level of the nucleic-acid-induced IL-23 mRNA expression. A correlation assay was carried out and confirmed the possible negative correlation between the nucleic-acid-induced IL-23 mRNA expression levels and the decrease upon PRINS overexpression, suggesting the need for sufficiently high levels of IL-23 mRNA for PRINS regulation to be detected. However, even in cases when the induction of IL-23 expression was sufficiently high, PRINS might act indirectly rather than by direct interaction with the IL-23 mRNA.

Although IL-23 in psoriasis is predominantly thought to be released by professional immune cells, our results indicated that this cytokine might originate from keratinocytes as well. Nucleic acids activate several PRRs in these cells, leading to the upregulation of several inflammatory molecules, including IL-23. Thus, keratinocyte-derived nucleic acids in the skin might also contribute to the development of psoriasis by elevating the IL-23 levels in keratinocytes through an autocrine mode. Taken together, our data suggest that individual differences in sensitivity toward the levels of nucleic acids in the tissue might lead to different IL-23 levels. These differences might in turn be related to the different reactions toward biological agents used in psoriasis treatment. Detailed analysis of these processes will help us to identify new biomarkers for the development of personalized treatments for the disease. 

## 4. Materials and Methods

### 4.1. Cell Culture

For these experiments, we used NHEKs isolated from skin samples retrieved from the Plastic Surgery Unit of our department after informed written consent was obtained from the volunteers. The experiments were approved by the Human Investigation Review Board of the University of Szeged (PSO-EDAFN-002, 23 February 2015, Szeged, Hungary), and investigations were carried out in accordance with the rules of the Helsinki Declaration. The isolation of the NHEKs from skin samples was described earlier [28]. Keratinocytes were grown in Keratinocyte-SFM Medium (Gibco, Thermo Fischer Scientific, Waltham, MA, USA), supplemented with EGF (epidermal growth factor), BPE (bovine pituitary extract), 1% L-glutamine and 1% antibiotic–antimycotic solution. The medium was changed every second day until the third passage.

### 4.2. Stimulation of the Cells

Third-passage NHEK cells were seeded into six-well plates at a density of 200,000 cells/mL. After 24 h, the medium was changed to supplement-free medium, and cells were transfected with 1 μg/mL poly deoxyadenylic acid–poly deoxythymidylic acid double-stranded homopolymer (poly(dA:dT)) (InvivoGen, San Diego, CA, USA) or with 0.666 μg/mL polyinosinic–polycytidylic acid (poly(I:C)) (Sigma Aldrich, Saint Louis, MO, USA) using the X-tremeGENE 9 transfection reagent (Roche Diagnostics, Basel, Switzerland), according to the manufacturer’s instructions. Cells were harvested at indicated time points.

For PRINS overexpression, the AK022045 cDNA sequence (Biological Research Center, National Institute of Technology and Evaluation, Chiba, Japan) was cloned into a pcDNA3.1(+) vector as described previously [14]. As a control, the empty pcDNA3.1(+) vector was used. One microgram of plasmid DNA was used for the transfection of the NHEK cells using the X-tremeGENE HP transfection reagent (Roche Diagnostics), according to the manufacturer’s instructions.

For siRNA-mediated gene silencing of PRRs, the ON-TARGETplus SMARTpool TLR3, RIG-1, IFIH-1, cGAS siRNAs or ON-TARGETplus Non-Targeting Pool (Dharmacon, Lafayette, CO, USA) constructs were used at a final concentration of 40 nM for transfection using the X-tremeGENE siRNA transfection reagent (Roche Diagnostics), according to the manufacturer’s instructions.

For inhibition experiments, NHEK cells were incubated 1 h prior to poly(dA:dT)/poly(I:C) transfection with specific inhibitors for NF-κB (Bay 11-7085, 10 μM; MedChemExpress, Monmouth Junction, NJ, USA), STAT-1 (Fludarabine, 10 μM; Sigma Aldrich), STAT-3 (Stattic, 5 μM; Sigma Aldrich), JNK (SP600125, 10 μM; Tocris Bioscience, Bristol, UK), MEK-1 (PD98059, 20 μM; Sigma Aldrich) and p38 (SB203580, 10 μM; Tocris Bioscience). Since all of the signal transduction pathway inhibitors were solved in DMSO (Sigma Aldrich) to different concentrations, the same amount of DMSO was applied as control.

### 4.3. RNA Isolation and RT-PCR

At indicated time points after transfection, cells were harvested in TRIzol^®^ Reagent (Invitrogen Corp., Carlsbad, CA, USA), and total RNA was isolated following the manufacturer’s instructions. Potential genomic DNA contamination was removed using the Turbo DNA-free Kit (Ambion, Thermo Fischer Scientific), according to the manufacturer’s instructions. During cDNA synthesis, 1 μg total RNA was reverse transcribed by the EvoScript Universal cDNA master mix (Roche Diagnostics). Real-time RT-PCR experiments were carried out with the TaqMan Gene expression Assay (PRINS: Hs03671803_s1; IL-23p19: Hs00900828_g1, GAPDH: Hs01548420_m1) (Thermo Fisher Scientific) using the qPCRBIO Probe Mix Lo-ROX (PCR Biosystem Ltd., London, UK) on a C1000 Touch Thermal Cycler (Bio-Rad Laboratories, Hercules, CA, USA). The expression of each gene was normalized to the GAPDH mRNA, and relative mRNA levels were calculated using the ∆∆Ct method.

### 4.4. In Silico Prediction of Interacting Sites

Using INTARNA [53], sequence complementarity between PRINS (AK022045) and the mRNA of IL-23 (NM_016584.3) was analyzed. INTARNA calculates the free-energy values of interaction based on predicted global and local structures of mRNAs. This in silico analysis revealed a binding site on the IL-23A mRNA that partly overlaps with the previously identified IL-6 binding sequences [14].

### 4.5. pmirGLO Dual-Luciferase miRNA Target Expression Vector

The sequence of the putative PRINS binding site of IL-23 cDNA that was identified in silico was synthesized and inserted into the pmirGLO vector (Promega, Madison, WI, USA) between the *Dra* I and *Xba* I restriction sites, resulting in the pmirGLO-IL23BS construct (Supplementary Figure S4). HEK293 cells were transiently transfected by the PRINSpcDNA3.1 or pmirGLO plasmid as controls. Cells were also co-transfected with 0.5 μg pmirGLO-IL23BS or empty pmirGLO plasmid together with the PRINSpcDNA3.1 vector. In each transfection experiment, 0.025 μg renilla-luciferase-expressing pGL4.75 hRluc/CMV (Promega) plasmid was used as internal control. Cells were harvested after 24 h, washed with PBS (phosphate-buffered saline) and lysed with passive lysis buffer (Biotium, Hayward, CA, USA), and luciferase activities were measured using the Firefly and Renilla Dual Luciferase Assay Kit (Biotium) and SYNERGY/HTX Multi-Mode reader (Bio Tek Instruments, Winooski, VT, USA), according to the manufacturer’s instructions. The luciferase activity derived from the pmirGLO plasmids was normalized to the renilla-luciferase activity.

### 4.6. Statistical Analysis

Experiments were carried out in duplicate with at least three biological repeats. For statistical analysis, a one-tailed, paired Student’s *t*-test was used with correction for multiple comparisons. The significance level was set at *p* ≤ 0.05. Pearson’s correlation calculation was used to analyze the correlation between IL-23 mRNA induction and PRINS-overexpression-mediated IL-23 mRNA depletion.

## Data Availability

Data is presented in the manuscript and is available upon request from the corresponding author.

## Supplementary Materials

The following are available online at https://www.mdpi.com/article/10.3390/ijms23010540/s1.
