# Supplementary material for: Psoriasis-Associated Inflammatory Conditions Induce IL-23 mRNA Expression in Normal Human Epidermal Keratinocytes
Source: Int J Mol Sci. 2022 Jan 4;23(1):540. doi: 10.3390/ijms23010540 (PMC8745281; doi:10.3390/ijms23010540)
Supplement: Supplementary file 1 [file ijms-23-00540-s001.zip › supplementary_table s1.pdf]

Supplementary Table S1. – List of genes included in the qPCR-array.

Gene Symbol	Alias	Refseq #	Official Full Name
AIM2	PYHIN4, OTTHUMP00000035296, interferon-inducible protein AIM2, RP11-520H16.3	NM_004833	absent in melanoma 2
CARD14	caspase recruitment domain protein 14, BIMP2, carma 2, CARD-containing MAGUK 2 protein, CARMA2, card-maguk protein 2, CARD-containing MAGUK protein 2, bcl10-interacting maguk protein 2, caspase recruitment domain-containing protein 14	NM_024110	caspase recruitment domain family, member 14
CARD18	ICEBERG, caspase recruitment domain-containing protein 18, ICEBERG caspase-1 inhibitor, caspase-1 inhibitor Iceberg, OTTHUMP00000232068, UNQ5804/PRO19611	NM_021571	caspase recruitment domain family, member 18
DCD	AIDD protein, MGC71930, diffusible survival/evasion peptide, HCAP, DCD-1, proteolysis inducing factor, DSEP, preproteolysin, AIDD, survival promoting peptide, PIF	NM_053283	dermcidin
GSDMC	melanoma-derived leucine zipper, extra-nuclear factor, OTTHUMP00000227707, melanoma-derived leucine zipper-containing extranuclear factor, MLZE, gasdermin-C	NM_031415	gasdermin C
GSDMD	DF5L, FKSG10, OTTHUMP00000228550, GSDMDC1, gasdermin-D, 1810036L03Rik, FLJ12150, OTTHUMP00000228551, gasdermin domain-containing protein 1, gasdermin domain containing 1, OTTHUMP00000228549	NM_001166237	gasdermin D
DEFB1	BD1, OTTHUMP00000159500, DEFB-1, HBD1, beta-defensin 1, beta-defensin-1, DEFB101, BD-1, MGC51822	NM_005218	defensin, beta 1
DEFB4A	SAP1, DEFB102, OTTHUMP00000159651, skin-antimicrobial peptide 1, beta-defensin 2, BD-2, DEFB2, defensin, beta 4, DEFB4, beta-defensin 4A, HBD-2, defensin, beta 2, DEFB-2	NM_004942	defensin, beta 4A
HMGB1	HMG1, OTTHUMP00000018198, HMG3, DKFZp686A04236, OTTHUMP00000018196, OTTHUMP00000018199, high-mobility group (nonhistone chromosomal) protein 1, OTTHUMP00000190859, high mobility group protein B1, SBP-1, OTTHUMP00000200117, HMG-1, high mobility group box 1, OTTHUMP00000018197, high-mobility group box 1, Amphoterin, high mobility group protein 1, OTTHUMP00000190860, Sulfoglucuronyl carbohydrate binding protein, RP11-550P23.1	NM_002128	high mobility group box 1
IFI16	PYHIN2, interferon-gamma induced protein IFI 16, OTTHUMP00000035197, IFNGIP1, interferon-inducible myeloid differentiation transcriptional activator, gamma-interferon-inducible protein 16, OTTHUMP00000035192, OTTHUMP00000035193, ifi-16	NM_001206567	interferon, gamma-inducible protein 16
IL19	MDA1, melanoma differentiation-associated protein-like protein, melanoma differentiation associated protein-like protein, IL-10C, interleukin-19, OTTHUMP00000216804, OTTHUMP00000034568, ZMDA1, NG.1, RP11-262N9.2	NM_013371	interleukin 19

IL36G	IL-1-epsilon, IL-1RP2, interleukin-1 family member 9, OTTHUMP00000203690, interleukin-1 homolog 1, IL-1 epsilon, IL1F9, interleukin 1 family, member 9, interleukin 1-related protein 2, IL-1-related protein 2, interleukin-1 epsilon, interleukin-36 gamma, IL1H1, IL-1F9, IL1RP2, IL-1(EPSILON), IL-1 related protein 2, IL1E, IL-1H1, OTTHUMP00000203689, UNQ2456/PRO5737	NM_019618	interleukin 36, gamma
IL36RN	FIL1, FIL1 delta, interleukin-1 family member 5, IL1RP3, IL1HY1, IL-1-related protein 3, IL1F5, interleukin 1 family, member 5 (delta), interleukin-36 receptor antagonist protein, interleukin-1-like protein 1, family of interleukin 1-delta, interleukin 1, delta, interleukin-1 HY1, IL-1F5 (IL-1HY1, FIL1-delta, IL-1RP3, IL-1L1, IL-1-delta), IL1F5 (Canonical product IL-1F5a), interleukin-1 delta, FIL1D, IL-1HY1, interleukin 1 family, member 5, interleukin-1 receptor antagonist homolog 1, IL-1ra homolog 1, OTTHUMP00000203698, OTTHUMP00000203697, FIL1(DELTA), OTTHUMP00000162015, IL-1L1, IL-1 related protein 3, IL-1ra homolog, IL1L1, MGC29840, IL-1 delta, UNQ1896/PRO4342	NM_012275	interleukin 36 receptor antagonist
IFIH1	Hlcd, RNA helicase-DEAD box protein 116, RIG-I-like receptor 2, interferon-induced with helicase C domain protein 1, helicard, CADM-140 autoantigen, IDDM19, MDA5, murabutide down-regulated protein, MGC133047, melanoma differentiation associated protein-5, melanoma differentiation-associated protein 5, interferon-induced helicase C domain-containing protein 1, helicase with 2 CARD domains, DEAD/H (Asp-Glu-Ala-Asp/His) box polypeptide, clinically amyopathic dermatomyositis autoantigen 140 kDa, OTTHUMP00000162935, MDA-5	NM_022168	interferon induced with helicase C domain 1
PYDC1	pyrin-only protein 1, POP1, PAAD-only protein 1, pyrin-domain containing protein 1, pyrin domain-containing protein 1, PYC1, OTTHUMP00000163243, pyrin domain containing 1, PAAD-only protein	NM_152901	PYD (pyrin domain) containing 1
DDX58	RIG-I, OTTHUMP00000115910, DKFZp686N19181, RIG-1, retinoic acid-inducible gene I, OTTHUMP00000045225, RIG-I-like receptor 1, DEAD/H (Asp-Glu-Ala-Asp/His) box polypeptide, RNA helicase, FLJ13599, retinoic acid-inducible gene 1 protein, retinoic acid inducible gene I, DEAD box protein 58, retinoic acid-inducible gene I protein, probable ATP-dependent RNA helicase DDX58, DEAD/H (Asp-Glu-Ala-Asp/His) box polypeptide RIG-I, RNA helicase RIG-I, OTTHUMP00000021185, RP11-334P12.2	NM_014314	DEAD (Asp-Glu-Ala-Asp) box polypeptide 58
POLR3A	RPC1, DNA-directed RNA polymerase III largest subunit, RNA polymerase III 155 kDa subunit, OTTHUMP00000060199, hRPC155, DNA-directed RNA polymerase III subunit RPC1, RPC155, RNA polymerase III subunit C1, RNA polymerase III subunit C160, DNA-directed RNA polymerase III subunit A, RNA polymerase III subunit RPC155-D, OTTHUMP00000019920	NM_007055	polymerase (RNA) III (DNA directed) polypeptide A, 155kDa
RNASE7	ribonuclease 7, skin-derived antimicrobial protein 2, MGC133220, OTTHUMP00000195134, RNase 7, SAP-2, UNQ2516/PRO6006	NM_032572	ribonuclease, RNase A family, 7

S100A7	PSOR1, OTTHUMP00000015328, S100 calcium-binding protein A7 (psoriasin 1), protein S100-A7, psoriasin 1, S100 calcium-binding protein A7, OTTHUMP00000015327, S100 calcium binding protein A7 (psoriasin 1), S100A7c	NM_002963	S100 calcium binding protein A7
S100A8	P8, OTTHUMP00000015330, 60B8AG, cystic fibrosis antigen, calprotectin L1L subunit, S100 calcium-binding protein A8 (calgranulin A), MRP-8, OTTHUMP00000015329, CP-10, MA387, urinary stone protein band A, calgranulin-A, MRP8, MIF, L1Ag, S100 calcium binding protein A8 (calgranulin A), CAGA, migration inhibitory factor-related protein 8, leukocyte L1 complex light chain, calgranulin A, S100 calcium-binding protein A8, protein S100-A8, CGLA, CFAG, NIF	NM_002964	S100 calcium binding protein A8
S100A9	S100 calcium-binding protein A9, CAGB, CGLB, migration inhibitory factor-related protein 14, S100 calcium binding protein A9 (calgranulin B), calprotectin L1H subunit, LIAG, protein S100-A9, NIF, S100 calcium-binding protein A9 (calgranulin B), MAC387, calgranulin B, leukocyte L1 complex heavy chain, MRP14, OTTHUMP00000015331, MIF, L1AG, MRP-14, calgranulin-B, 60B8AG, P14, CFAG	NM_002965	S100 calcium binding protein A9
PYDC2	LOC152138, OTTHUMP00000225036, pyrin-only protein 2, pyrin domain-containing protein 2, POP2	NM_001083308	pyrin domain containing 2
SOCS1	JAB, SSI-1, Tec-interacting protein 3, OTTHUMP00000160221, CIS1, SSI1, STAT-induced STAT inhibitor 1, JAK-binding protein, STAT induced SH3 protein 1, cytokine-inducible SH2 protein 1, TIP-3, SOCS-1, JAK binding protein, CISH1, TIP3	NM_003745	suppressor of cytokine signaling 1
TMEM173	LOC340061, OTTHUMP00000159544, endoplasmic reticulum IFN stimulator, stimulator of interferon genes, mitochondrial mediator of IRF3 activation, N-terminal methionine-proline-tyrosine-serine plasma membrane tetraspanner, hSTING, mediator of IRF3 activation, OTTHUMP00000223443, OTTHUMP00000223442, endoplasmic reticulum interferon stimulator, hypothetical protein LOC340061, stimulator of interferon genes protein, hMITA	NM_198282	transmembrane protein 173
TLR3	CD283, OTTHUMP00000218073	NM_003265	toll-like receptor 3
TLR7	OTTHUMP00000022919, toll-like receptor 7-like, UNQ248/PRO285	NM_016562	toll-like receptor 7
OAS2	P69, (2 '-5 ')oligo(A) synthetase 2, 2'-5'-oligoadenylate synthetase 2 (69-71 kD), 2-5A synthetase 2, (2-5')oligo(A) synthetase 2, MGC78578, 2'-5'-oligoadenylate synthase 2, 2'5' oligoadenylate synthetase 2, p69 OAS / p71 OAS, 2 '5 ' oligoadenylate synthetase 2, (2'-5')oligo(A) synthetase 2, (2-5')oligo(A) synthase 2, 2 '-5 '-oligoadenylate synthetase 2, 2'-5'-oligoadenylate synthetase 2, 2'-5'oligoadenylate synthetase 2, 2'-5'-oligoadenylate synthetase 2 (69-71 kD), 2-5A synthase 2, 2 '-5 '-oligoadenylate synthetase 2 (69-71 kD), p69OAS / p71OAS, 2'-5'-oligoadenylate synthetase 2, (2'-5')oligo(A) synthetase 2, 2'5' oligoadenylate synthetase 2	NM_001032731	2'-5'-oligoadenylate synthetase 2, 69/71kDa

BPI	bK2308N23.1 (bactericidal/permeability-increasing protein), CAP 57, OTTHUMP00000030963, BPI fold containing family D, member 1, OTTHUMP00000030962, bactericidal permeability-increasing protein, recombinant BPI holoprotein, rBPI, RP13-218F6_A.1	NM_001725	bactericidal/permeability-increasing protein
CAMP	LL37, CAP-18, hCAP-18, OTTHUMP00000164805, FALL39, FALL-39, CAP18, HSD26, 18 kDa cationic antimicrobial protein	NM_004345	cathelicidin antimicrobial peptide
CASP1	caspase 1, interleukin-1 beta convertase, interleukin-1 beta-converting enzyme, IL-1 beta-converting enzyme, OTTHUMP00000232031, OTTHUMP00000045728, OTTHUMP00000232030, caspase 1, apoptosis-related cysteine peptidase (interleukin 1, beta, convertase), caspase 1, apoptosis-related cysteine protease (interleukin 1, beta, convertase), IL1BC, interleukin 1-beta convertase, interleukin 1-B converting enzyme, P45, IL-1BC, CASP1 nirs variant 1, IL1B-convertase, CASP-1, OTTHUMP00000232036, OTTHUMP00000232033, OTTHUMP00000232032, caspase-1, interleukin 1, beta, convertase, OTTHUMP00000232034, OTTHUMP00000232029, ICE	NM_001223, NM_033292, NM_033293, NM_033294, NM_033295	caspase 1, apoptosis-related cysteine peptidase
CASP8	MACH, procaspase-8L, MORT1-associated ced-3 homolog, caspase, apoptotic cysteine protease, OTTHUMP00000163717, caspase 8, apoptotic protease Mch-5, FLICE, caspase 8, apoptosis-related cysteine protease, FADD-homologous ICE/CED-3-like protease, OTTHUMP00000206552, OTTHUMP00000163720, Mch5 isoform alpha, MACH-beta-1/2/3/4 protein, OTTHUMP00000163718, MACH-alpha-1/2/3 protein, cysteine protease, OTTHUMP00000165062, CAP4, OTTHUMP00000163719, OTTHUMP00000165064, caspase-8, MGC78473, MCH5, FADD-like ICE, OTTHUMP00000165063, procaspase-8, OTTHUMP00000206581, ICE-like apoptotic protease 5, OTTHUMP00000206557	NM_001080124	caspase 8, apoptosis-related cysteine peptidase
CCL17	small inducible cytokine A17, small-inducible cytokine A17, ABCD-2, OTTHUMP00000164673, small inducible cytokine subfamily A (Cys-Cys), member 17, MGC138273, thymus and activation-regulated chemokine, CC chemokine TARC, MGC138271, A-152E5.3, C-C motif chemokine 17, SCYA17, T cell-directed CC chemokine, TARC	NM_002987	chemokine (C-C motif) ligand 17
CCL3	LD78 alpha beta, Small inducible cytokine A3, OTTHUMP00000163953, SIS-beta, MIP1A, small-inducible cytokine A3, PAT 464.1, SCYA3, C-C motif chemokine 3, LD78ALPHA, tonsillar lymphocyte LD78 alpha protein, MIP-1-alpha, G0S19-1, macrophage inflammatory protein 1-alpha, G0/G1 switch regulatory protein 19-1, small inducible cytokine A3 (homologous to mouse Mip-1a), MIP-1A	NM_002983	chemokine (C-C motif) ligand 3

CCL5	small inducible cytokine A5, D17S136E, TCP228, regulated upon activation, normally T-expressed, and presumably secreted, eosinophil chemotactic cytokine, beta-chemokine RANTES, SIS-delta, RANTES, t cell-specific protein P228, small inducible cytokine subfamily A (Cys-Cys), member 5, T-cell-specific protein RANTES, chemokine ligand 5, T-cell specific RANTES protein, SCYA5, small inducible cytokine A5 (RANTES), T-cell specific protein p288, OTTHUMP00000197106, SISd, C-C motif chemokine 5, beta-chemokine RANTES precursor, small-inducible cytokine A5, OTTHUMP00000163880, MGC17164, eoCP	NM_002985	chemokine (C-C motif) ligand 5
CCL2	small inducible cytokine A2, monocyte chemoattractant protein 1, small-inducible cytokine A2, small inducible cytokine A2 (monocyte chemotactic protein 1, homologous to mouse Sig-je), OTTHUMP00000163799, monocyte chemotactic protein 1, HSMCR30, small inducible cytokine subfamily A (Cys-Cys), member 2, MCP-1, SCYA2, MCAF, SMC-CF, GDCF-2 HC11, HC11, monocyte chemotactic protein 1, homologous to mouse Sig-je, MGC9434, monocyte secretory protein JE, C-C motif chemokine 2, GDCF-2, MCP1, monocyte chemotactic and activating factor, monocyte chemoattractant protein-1	NM_002982	chemokine (C-C motif) ligand 2
CCL20	CKb4, small-inducible cytokine A20, SCYA20, small inducible cytokine subfamily A (Cys-Cys), member 20, C-C motif chemokine 20, ST38, exodus-1, liver and activation-regulated chemokine, macrophage inflammatory protein 3 alpha, MIP-3-alpha, OTTHUMP00000164245, OTTHUMP00000204113, beta chemokine exodus-1, LARC, MIP-3a, beta-chemokine exodus-1, CC chemokine LARC, MIP3A	NM_001130046	chemokine (C-C motif) ligand 20
CXCL1	GRO1, growth-regulated alpha protein, GRO1 oncogene (melanoma growth stimulating activity, alpha), GROa, OTTHUMP00000219186, fibroblast secretory protein, MGSA alpha, chemokine (C-X-C motif) ligand 1, MGSA, C-X-C motif chemokine 1, SCYB1, MGSA-a, GRO-alpha(1-73), GRO1 oncogene (melanoma growth-stimulating activity), melanoma growth stimulatory activity alpha, neutrophil-activating protein 3, NAP-3, GRO1, GROa, SCYB1	NM_001511	chemokine (C-X-C motif) ligand 1 (melanoma growth stimulating activity, alpha)
CXCL2	GRO2 oncogene, MGSA-b, MGSA beta, SCYB2, MIP2-alpha, gro-beta, OTTHUMP00000219185, MIP-2a, growth-regulated protein beta, CINC-2a, C-X-C motif chemokine 2, MIP2, GRO2, MIP2A, GROb, melanoma growth stimulatory activity beta, macrophage inflammatory protein 2-alpha, GRO2, GROb, SCYB2	NM_002089	chemokine (C-X-C motif) ligand 2
FADD	Fas-associated via death domain, Fas-associating protein with death domain, FAS-associated death domain protein, Fas-associating protein, MORT1, growth-inhibiting gene 3 protein, OTTHUMP00000235551, MGC8528, mediator of receptor induced toxicity, mediator of receptor-induced toxicity, GIG3, protein FADD, Fas-associating death domain-containing protein	NM_003824	Fas (TNFRSF6)-associated via death domain

IFNA1	IFN-alpha 1b, IFL, IFNA13, OTTHUMP00000021142, IFN, MGC138505, IFN-ALPHA, leIF D, MGC138207, MGC138507, interferon alpha 1b, interferon alpha-D, IFN-alpha-1/13, interferon alpha-1/13, interferon-alpha1, OTTHUMP00000045110, IFNA@	NM_024013	interferon, alpha 1
IFNB1	IFB, fibroblast interferon, IFN-beta, IFF, IFNB, interferon beta, MGC96956, OTTHUMP00000021131, RP11-113D19.1	NM_002176	interferon, beta 1, fibroblast
IL12A	CLMF, IL-12 subunit p35, IL35 subunit, OTTHUMP00000213703, interleukin-12 alpha chain, NF cell stimulatory factor chain 1, CLMF p35, natural killer cell stimulatory factor 1, 35 kD subunit, NKSF1, OTTHUMP00000213704, OTTHUMP00000213705, interleukin-12 alpha chain precursor, interleukin 12, p35, NK cell stimulatory factor chain 1, NFSK, IL-12A, cytotoxic lymphocyte maturation factor 1, p35, interleukin 12A, IL-12, subunit p35, interleukin-12 subunit alpha, cytotoxic lymphocyte maturation factor 35 kDa subunit, interleukin 12A (natural killer cell stimulatory factor 1, cytotoxic lymphocyte maturation factor 1,	NM_000882	interleukin 12A
IL12B	interleukin 12B, CLMF p40, NK cell stimulatory factor chain 2, IL-12 subunit p40, CLMF, cytotoxic lymphocyte maturation factor 2, p40, cytotoxic lymphocyte maturation factor 40 kDa subunit, IL12, subunit p40, natural killer cell stimulatory factor, 40 kD subunit, OTTHUMP00000160820, natural killer cell stimulatory factor-2, CLMF2, interleukin 12, p40, NKSF, interleukin-12 subunit beta, IL-12B, interleukin-12 beta chain, NKSF2, interleukin 12B (natural killer cell stimulatory factor 2, cytotoxic lymphocyte maturation factor 2,	NM_002187	interleukin 12B
IL18	IGIF, OTTHUMP00000234749, interferon-gamma-inducing factor, MGC12320, interferon gamma-inducing factor, IFN-gamma-inducing factor, iboctadekin, IL-1 gamma, IL-1g, IL1F4, interleukin-1 gamma, OTTHUMP00000234751, IL-18, OTTHUMP00000234750, interleukin 18, interleukin-18, interleukin 18 (interferon-gamma-inducing factor)	NM_001243211	interleukin 18
CX3CL1	NTN, C-X3-C motif chemokine 1, CX3C membrane-anchored chemokine, SCYD1, CXC3C, ABCD-3, CXC3, neurotactin, NTT, small inducible cytokine subfamily D (Cys-X3-Cys), member-1, small-inducible cytokine D1, C3Xkine, OTTHUMP00000164674, small inducible cytokine subfamily D (Cys-X3-Cys), member 1 (fractalkine, neurotactin), small inducible cytokine subfamily D (Cys-X3-Cys), member 1, fractalkine, A-152E5.2	NM_002996	chemokine (C-X3-C motif) ligand 1
IL1B	IL-1, IL-1 beta, IL1-BETA, IL1F2, interleukin-1 beta, OTTHUMP00000203693, catabolin, pro-interleukin-1-beta, OTTHUMP00000203694, OTTHUMP00000203695, OTTHUMP00000162031, preinterleukin 1 beta	NM_000576	interleukin 1, beta
IL6	HGF, BSF-2, OTTHUMP00000198489, CTL differentiation factor, B cell stimulatory factor-2, OTTHUMP00000158544, OTTHUMP00000198490, HSF, B-cell stimulatory factor 2, BSF2, B-cell differentiation factor, IFNB2, IFN-beta-2, interleukin 6, CDF, interleukin-6, interleukin BSF-2, hybridoma	NM_000600	interleukin 6

	growth factor, interferon beta-2, OTTHUMP00000198486, IL-6, interferon, beta 2		
CXCL10	C7, small-inducible cytokine B10, INP10, OTTHUMP00000219215, 10 kDa interferon gamma-induced protein, gamma-IP10, crg-2, small inducible cytokine subfamily B (Cys-X-Cys), member 10, IFI10, protein 10 from interferon (gamma)-induced cell line, interferon-inducible cytokine IP-10, IP-10, SCYB10, gamma IP10, C-X-C motif chemokine 10, mob-1, gIP-10, small inducible cytokine B10	NM_001565	chemokine (C-X-C motif) ligand 10
CXCL8	K60, chemokine (C-X-C motif) ligand 8, lymphocyte derived neutrophil activating peptide, LUCT, C-X-C motif chemokine 8, OTTHUMP00000199824, protein 3-10C, MDNCF, TSG-1, monocyte-derived neutrophil chemotactic factor, neutrophil-activating factor, MONAP, LECT, interleukin-8, AMCF-I, GCP1, LUCT/interleukin-8, CXC chemokine ligand 8, granulocyte chemotactic protein 1, emoctakin, T-cell chemotactic factor, NAP1, CXCL8, b-ENAP, lung giant cell carcinoma-derived chemotactic protein, LYNAP, SCYB8, small inducible cytokine subfamily B, member 8, GCP-1, monocyte derived neutrophil-activating protein, alveolar macrophage chemotactic factor I, NAP-1, neutrophil-activating peptide 1, T cell chemotactic factor, IL-8, tumor necrosis factor-induced gene 1, NAF, OTTHUMP00000199825, neutrophil-activating protein 1, 3-10C, beta endothelial cell-derived neutrophil activating peptide, lymphocyte-derived neutrophil-activating factor, beta-thromboglobulin-like protein, monocyte-derived neutrophil-activating peptide, interleukin 8, IL8	NM_000584	chemokine (C-X-C motif) ligand 8
LBP	lipopolysaccharide-binding protein, MGC22233, OTTHUMP00000030965, LPS-binding protein, BPI fold containing family D, member 2	NM_004139	lipopolysaccharide binding protein
CXCL9	CMK, small-inducible cytokine B9, C-X-C motif chemokine 9, monokine induced by interferon-gamma, SCYB9, gamma-interferon-induced monokine, monokine induced by gamma interferon, MIG, OTTHUMP00000219216, small inducible cytokine B9, crg-10, Humig	NM_002416	chemokine (C-X-C motif) ligand 9
LTF	HLF2, OTTHUMP00000209781, neutrophil lactoferrin, OTTHUMP00000209779, lactoferrin, GIG12, talalactoferrin, growth-inhibiting protein 12, OTTHUMP00000209780, kaliocin-1, lactoferricin, epididymis luminal protein 110, lactoferroxin	NM_001199149	lactotransferrin
IFNA2	interferon alpha A, interferon alpha 2a, INFA2, leIF A, interferon alpha 2b, cytokine, IFNA, interferon alpha-A, OTTHUMP00000021143, alpha-2a interferon, IFN-alpha-2, MGC125764, MGC125765, interferon alpha-2, RP11-354P17.2	NM_000605	interferon, alpha 2
LYZ	lysozyme, 1,4-beta-N-acetylmuramidase C, renal amyloidosis, lysozyme C, c-type lysozyme	NM_000239	lysozyme
IFNG	IFG, OTTHUMP00000240111, immune interferon, IFI, IFN-gamma, interferon gamma	NM_000619	interferon, gamma

IL10	cytokine synthesis inhibitory factor, OTTHUMP00000034567, TGIF, IL-10, IL10A, CSIF, MGC126451, T-cell growth inhibitory factor, interleukin-10, MGC126450, RP11-262N9.1	NM_000572	interleukin 10
IL17F	ML1, interleukin-24, cytokine ML-1, IL-17F, OTTHUMP00000016602, interleukin-17F, ML-1, IL-24, mutant IL-17F	NM_052872	interleukin 17F
IL1A	IL1A (IL1F1), IL-1A, interleukin-1 alpha, OTTHUMP00000203692, OTTHUMP00000161990, hematopoietin-1, preinterleukin 1 alpha, IL1F1, IL-1 alpha, IL1, IL1-ALPHA, pro-interleukin-1-alpha	NM_000575	interleukin 1, alpha
NAIP	neuronal apoptosis inhibitory protein, psi neuronal apoptosis inhibitory protein, baculoviral IAP repeat-containing protein 1, OTTHUMP00000125255, BIRC1, psiNAIP, OTTHUMP00000226926, NLR family, BIR domain containing 1, OTTHUMP00000226927, NAIP, baculoviral IAP repeat-containing 1, nucleotide-binding oligomerization domain, leucine rich repeat and BIR domain containing 1, OTTHUMP00000226928	NM_004536	NLR family, apoptosis inhibitory protein
IL1RN	IRAP, IL1RA, MGC10430, type II interleukin-1 receptor antagonist, OTTHUMP00000203730, OTTHUMP00000162024, OTTHUMP00000162022, IL1F3, IL1RN (IL1F3), intracellular IL-1 receptor antagonist type II, intracellular interleukin-1 receptor antagonist (icIL-1ra), IL-1ra3, interleukin-1 receptor antagonist protein, OTTHUMP00000162023, IL1 inhibitor, OTTHUMP00000203731, ICIL-1RA	NM_000577	interleukin 1 receptor antagonist
NLRC4	CLAN, CLANB, CARD12, OTTHUMP00000201170, OTTHUMP00000201173, ICE-protease activating factor, nucleotide-binding oligomerization domain, leucine rich repeat and CARD domain containing 4, CLANA, OTTHUMP00000201172, CARD, LRR, and NACHT-containing protein, NLR family CARD domain-containing protein 4, caspase recruitment domain protein 12, CLANC, NOD-like receptor C4, caspase recruitment domain-containing protein 12, caspase recruitment domain family, member 12, CLAND, ice protease-activating factor, CLAN1, OTTHUMP00000201171, IPAF, UNQ6189/PRO20215	NM_001199138	NLR family, CARD domain containing 4
NLRP1	CARD7, NACHT, LRR and PYD containing protein 1, systemic lupus erythematosus, vitiligo-related 1, DEFCAP-L/S, NAC-alpha/beta/gamma/delta, DKFZp586O1822, NACHT, leucine rich repeat and PYD (pyrin domain) containing 1, NACHT, leucine rich repeat and PYD containing 1, death effector filament-forming Ced-4-like apoptosis protein, nucleotide-binding domain and caspase recruitment domain, NACHT, LRR and PYD domains-containing protein 1, PP1044, DEFCAP, nucleotide-binding oligomerization domain, leucine rich repeat and pyrin domain containing 1, KIAA0926, caspase recruitment domain protein 7, caspase recruitment domain-containing protein 7, NALP1	NM_001033053	NLR family, pyrin domain containing 1
IL22	interleukin 21, zcyto18, ILTIF, interleukin-22, cytokine Zcyto18, IL-10-related T-cell-derived inducible factor, TIFIL-23, IL-21, MGC79382, IL-D110, IL-TIF, IL-22, TIFa, OTTHUMP00000240117, IL-10-related T-cell-derived-inducible factor, IL21, MGC79384, UNQ3099/PRO10096	NM_020525	interleukin 22

NLRP3	FCU, cryopyrin, cold autoinflammatory syndrome 1 protein, OTTHUMP00000230825, PYRIN-containing APAF1-like protein 1, NACHT, LRR and PYD domains-containing protein 3, OTTHUMP00000038409, nucleotide-binding oligomerization domain, leucine rich repeat and pyrin domain containing 3, caterpiller protein 1.1, CIAS1, AGTAVPRL, Muckle-Wells syndrome, OTTHUMP00000038410, OTTHUMP00000038411, chromosome 1 open reading frame 7, NACHT, LRR and PYD containing protein 3, NALP3, MWS, NACHT domain-, leucine-rich repeat-, and PYD-containing protein 3, AII/AVP, OTTHUMP00000038408, angiotensin/vasopressin receptor AII/AVP-like, AII/AVP receptor-like, FCAS, cold autoinflammatory syndrome 1, PYPAF1, C1orf7	NM_001079821	NLR family, pyrin domain containing 3
IL23A	interleukin 23 p19 subunit, interleukin-23 subunit alpha, MGC79388, IL-23-A, P19, IL23P19, IL-23p19, interleukin-23 subunit p19, JKA3 induced upon T-cell activation, IL-23A, IL-23 subunit alpha, IL-23, SGRF, interleukin-six, G-CSF related factor, UNQ2498/PRO5798	NM_016584	interleukin 23, alpha subunit p19
NOD1	caspase recruitment domain 4, caspase recruitment domain family, member 4, nucleotide-binding oligomerization domain-containing protein 1, OTTHUMP00000158742, NLR family, CARD domain containing 1, CARD4, nucleotide-binding oligomerization domain, leucine rich repeat and CARD domain containing 1, caspase recruitment domain-containing protein 4, NOD1	NM_006092	nucleotide-binding oligomerization domain containing 1
IL24	C49A, IL-4-induced secreted protein, interleukin-24, melanocyte-associated Mda-7, Mob-5, IL-24 splice variant delE3, melanoma differentiation-associated gene 7 protein, suppression of tumorigenicity 16 (melanoma differentiation), mda-7, MDA7, IL-24, ST16, OTTHUMP00000034621, OTTHUMP00000216789, OTTHUMP00000034620, FISP, suppression of tumorigenicity 16 protein, IL-24 splice variant delE5, melanoma differentiation association protein 7, IL10B, OTTHUMP00000216790	NM_001185156	interleukin 24
NOD2	CD, LRR-containing protein, BLAU, NOD2 protein, Arthrocutaneouveal granulomatosis (Blau syndrome), OTTHUMP00000164224, NOD2, nucleotide-binding oligomerization domain 2, IBD1, OTTHUMP00000236088, NOD2B, PSORAS1, ACUG, caspase recruitment domain protein 15, nucleotide-binding oligomerization domain, leucine rich repeat and CARD domain containing 2, NOD-like receptor C2, nucleotide-binding oligomerization domain-containing protein 2, inflammatory bowel disease protein 1, NLR family, CARD domain containing 2, caspase recruitment domain family, member 15, caspase recruitment domain-containing protein 15, CARD15	NM_022162	nucleotide-binding oligomerization domain containing 2
IL27	p28, interleukin-27 subunit alpha, IL30, MGC71873, IL-27 p28 subunit, OTTHUMP00000122516, IL-27, IL-27 subunit alpha, interleukin 30, IL27-A, IL27p28, IL-27-A, interleukin-30	NM_145659	interleukin 27

PYCARD	caspase recruitment domain protein 5, OTTHUMP00000163240, hASC, CARD5, caspase recruitment domain-containing protein 5, OTTHUMP00000163239, TMS1, PYD and CARD domain-containing protein, MGC10332, target of methylation-induced silencing 1, target of methylation-induced silencing-1, apoptosis-associated speck-like protein containing a CARD, ASC, TMS1, ASC	NM_013258	PYD and CARD domain containing
LIF	CDF, differentiation-stimulating factor, MLPLI, differentiation-inducing factor, human interleukin in DA cells, differentiation inhibitory activity, melanoma-derived LPL inhibitor, D-FACTOR, cholinergic differentiation factor, differentiation stimulating factor, leukemia inhibitory factor, D factor, OTTHUMP00000198698, hepatocyte-stimulating factor III, HILDA	NM_002309	leukemia inhibitory factor
LTA	lymphotoxin alpha, OTTHUMP00000165897, tumor necrosis factor ligand superfamily member 1, cytokine, TNFB, lymphotoxin-alpha, LT, LT-alpha, tumor necrosis factor beta, TNF-beta, OTTHUMP00000037613, OTTHUMP00000029184, TNFSF1, OTTHUMP00000037612, lymphotoxin A, DAMA-25N12.13-004, TNF superfamily, member 1	NM_000595	lymphotoxin alpha
SLPI	ALP, secretory leukocyte protease inhibitor (antileukoproteinase), mucus proteinase inhibitor, secretory leukocyte protease inhibitor, HUSI-I, protease inhibitor WAP4, ALK1, BLPI, HUSI, seminal proteinase inhibitor, WAP four-disulfide core domain protein 4, WFDC4, MPI, HUSI-1, OTTHUMP00000031775, WAP four-disulfide core domain 4, antileukoproteinase, WAP4	NM_003064	secretory leukocyte peptidase inhibitor
LTB	OTTHUMP00000038214, p33, cytokine, TNFC, lymphotoxin-beta, tumor necrosis factor ligand superfamily member 3, TNF-C, tumor necrosis factor C, TNFSF3, LT-beta, OTTHUMP00000215145, OTTHUMP00000029185, DAAP-90L16.4	NM_002341	lymphotoxin beta (TNF superfamily, member 3)
MIF	phenylpyruvate tautomerase, GIF, glycosylation-inhibiting factor, L-dopachrome isomerase, OTTHUMP00000198465, macrophage migration inhibitory factor, MMIF, L-dopachrome tautomerase, GLIF	NM_002415	macrophage migration inhibitory factor (glycosylation-inhibiting factor)
TLR2	TIL4, toll/interleukin 1 receptor-like 4, toll/interleukin-1 receptor-like protein 4, CD282	NM_003264	toll-like receptor 2
TLR4	TOLL, hToll, homolog of Drosophila toll, OTTHUMP00000022807, CD284	NM_003266	toll-like receptor 4
TLR5	TIL3, Toll/interleukin-1 receptor-like protein 3, MGC126431, FLJ10052, OTTHUMP00000036040, MGC126430, OTTHUMP00000036039, RP11-239E10.1, systemic lupus erythematosus susceptibility 1	NM_003268	toll-like receptor 5
TLR9	CD289, OTTHUMP00000212618, UNQ5798/PRO19605	NM_017442	toll-like receptor 9
TNF	OTTHUMP00000037669, tumor necrosis factor alpha, TNF-alpha, TNFA, OTTHUMP00000029281, TNF, macrophage-derived, cachectin, APC1 protein, TNF superfamily, member 2, TNF-a, TNFSF2, tumor necrosis factor-alpha, tumor necrosis factor ligand superfamily member 2, TNF, monocyte-derived, DIF, DADB-70P7.1	NM_000594	tumor necrosis factor

TRAF6	RNF85, E3 ubiquitin-protein ligase TRAF6, OTTHUMP00000232773, TNF receptor-associated factor 6, MGC:3310, OTTHUMP00000232772, RING finger protein 85, interleukin-1 signal transducer	NM_004620	TNF receptor-associated factor 6, E3 ubiquitin protein ligase
VEGFA	VEGFA, OTTHUMP00000224109, OTTHUMP00000016487, vascular endothelial growth factor isoform VEGF165, OTTHUMP00000224153, OTTHUMP00000224425, OTTHUMP00000224107, vascular endothelial growth factor 165b, VPF, OTTHUMP00000016488, MGC70609, OTTHUMP00000224426, VEGF, OTTHUMP00000224108, OTTHUMP00000224427, OTTHUMP00000224430, OTTHUMP00000224428, OTTHUMP00000165986, OTTHUMP00000165985, OTTHUMP00000224429, OTTHUMP00000224423, OTTHUMP00000165987, growth factor, vascular permeability factor, OTTHUMP00000224424, OTTHUMP00000224154, RP1-261G23.1	NM_001025366	vascular endothelial growth factor A
XCL1	LTN, small-inducible cytokine C1, XC chemokine ligand 1, LPTN, SCM-1-alpha, OTTHUMP00000032498, SCM1, SCYC1, lymphotaxin, c motif chemokine 1, SCM-1a, SCM-1, small inducible cytokine subfamily C, member 1 (lymphotactin), single cysteine motif 1a, OTTHUMP00000060404, lymphotactin, region containing small inducible cytokine subfamily C, member 1 (lymphotactin); small inducible cyt, ATAC, cytokine SCM-1	NM_002995	chemokine (C motif) ligand 1
ZBP1	DLM1, DLM-1, chromosome 20 open reading frame 183, DNA-dependent activator of IFN-regulatory factors, OTTHUMP00000237854, OTTHUMP00000031373, DNA-dependent activator of interferon regulatory factors, tumor stroma and activated macrophage protein DLM-1, C20ORF183, OTTHUMP00000174290, Z-DNA-binding protein 1, DNA-dependent activator of IRFs, RP4-718J7.1	NM_001160417	Z-DNA binding protein 1
ACTB	beta actin, OTTHUMP00000024886, beta cytoskeletal actin, actin, cytoplasmic 1, PS1TP5-binding protein 1, beta-actin	NM_001101	actin, beta
B2M	beta chain of MHC class I molecules, beta-2-microglobin, OTTHUMP00000161912, CDABP0092	NM_004048	beta-2-microglobulin
GAPDH	G3PD, aging-associated gene 9 protein, MGC88685, OTTHUMP00000174434, OTTHUMP00000174430, 3' end, peptidyl-cysteine S-nitrosylase GAPDH, OTTHUMP00000174431, OTTHUMP00000174432, glyceraldehyde 3-phosphate dehydrogenase, GAPDH, GAPD, CDABP0047, epididymis secretory sperm binding protein Li 162eP	NM_001256799	glyceraldehyde-3-phosphate dehydrogenase
HPRT1	HPRT, hypoxanthine phosphoribosyltransferase 1 (Lesch-Nyhan syndrome), HGPRT, OTTHUMP00000024061, HGPRTase, hypoxanthine phosphoribosyltransferase 1, hypoxanthine-guanine phosphoribosyltransferase, Lesch-Nyhan syndrome	NM_000194	hypoxanthine phosphoribosyltransferase 1
RPLP0	P0, L10E, PRLP0, MGC111226, MGC88175, 60S ribosomal protein L10E, ribosomal protein P0, acidic ribosomal phosphoprotein P0, RPP0, 60S acidic ribosomal protein P0	NM_001002	ribosomal protein, large, P0
GDC	Human Genomic DNA contamination		
PPC	Positive PCR Control		

RTC	Reverse Transcription Control		
