# Supplementary material for: Psoriasis-Associated Inflammatory Conditions Induce IL-23 mRNA Expression in Normal Human Epidermal Keratinocytes
Source: Int J Mol Sci. 2022 Jan 4;23(1):540. doi: 10.3390/ijms23010540 (PMC8745281; doi:10.3390/ijms23010540)
Supplement: Supplementary file 1 [file ijms-23-00540-s001.zip › ¿▓j-Psoriasis associated _supplementary figures-mod 2021 12 21.pptx]

## Slide 1
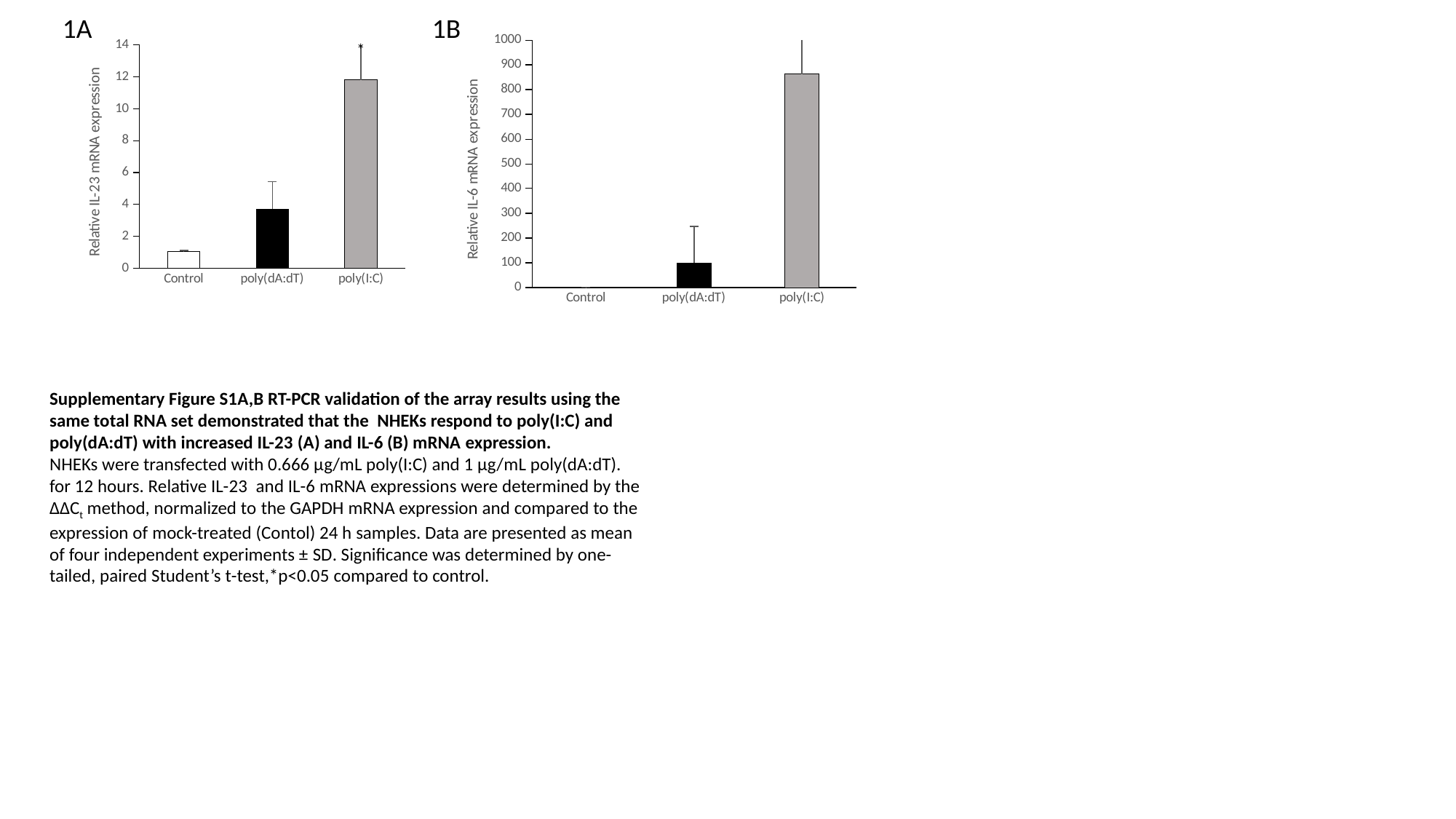

1A
1B
### Chart
| Category | 24h |
|---|---|
| Control | 1.045320514990466 |
| poly(dA:dT) | 3.733086057858589 |
| poly(I:C) | 11.806962221339084 |
### Chart
| Category | 24h |
|---|---|
| Control | 0.7967794574975666 |
| poly(dA:dT) | 99.08635199509162 |
| poly(I:C) | 862.606779637105 |Supplementary Figure S1A,B RT-PCR validation of the array results using the same total RNA set demonstrated that the NHEKs respond to poly(I:C) and poly(dA:dT) with increased IL-23 (A) and IL-6 (B) mRNA expression.
NHEKs were transfected with 0.666 μg/mL poly(I:C) and 1 μg/mL poly(dA:dT). for 12 hours. Relative IL-23 and IL-6 mRNA expressions were determined by the ∆∆Ct method, normalized to the GAPDH mRNA expression and compared to the expression of mock-treated (Contol) 24 h samples. Data are presented as mean of four independent experiments ± SD. Significance was determined by one-tailed, paired Student’s t-test,*p<0.05 compared to control.

## Slide 2
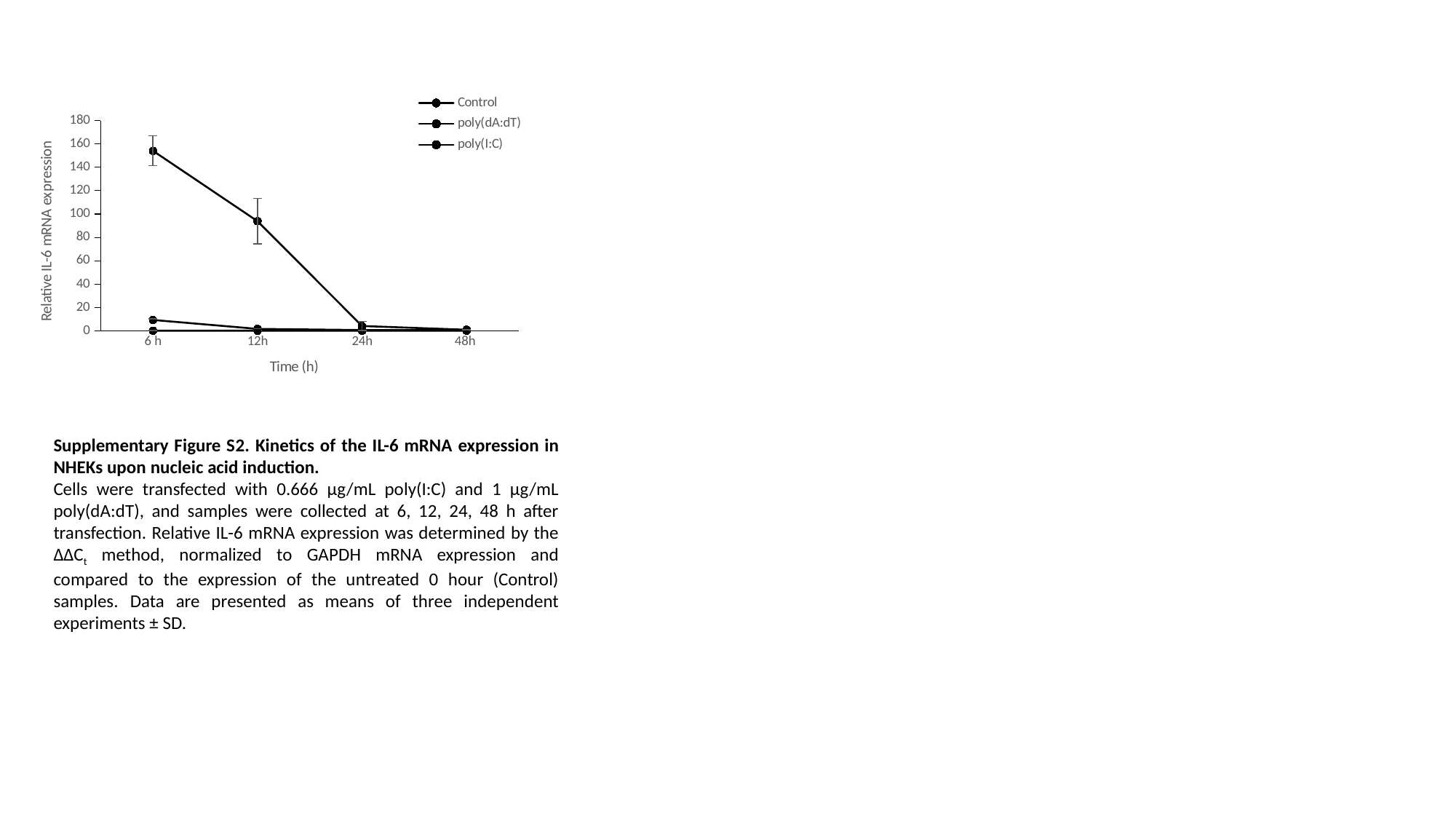

### Chart
| Category | Control | poly(dA:dT) | poly(I:C) |
|---|---|---|---|
| 6 h | 0.09170578944850347 | 9.28327399324661 | 154.12694556329865 |
| 12h | 0.08218036299939938 | 1.6440088142430769 | 93.87096365280344 |
| 24h | 0.0794237358809835 | 0.6313193490114979 | 4.08589153764988 |
| 48h | 0.06904731950349408 | 0.5896974304984357 | 0.8975615966211241 |Supplementary Figure S2. Kinetics of the IL-6 mRNA expression in NHEKs upon nucleic acid induction.
Cells were transfected with 0.666 μg/mL poly(I:C) and 1 μg/mL poly(dA:dT), and samples were collected at 6, 12, 24, 48 h after transfection. Relative IL-6 mRNA expression was determined by the ∆∆Ct method, normalized to GAPDH mRNA expression and compared to the expression of the untreated 0 hour (Control) samples. Data are presented as means of three independent experiments ± SD.

## Slide 3
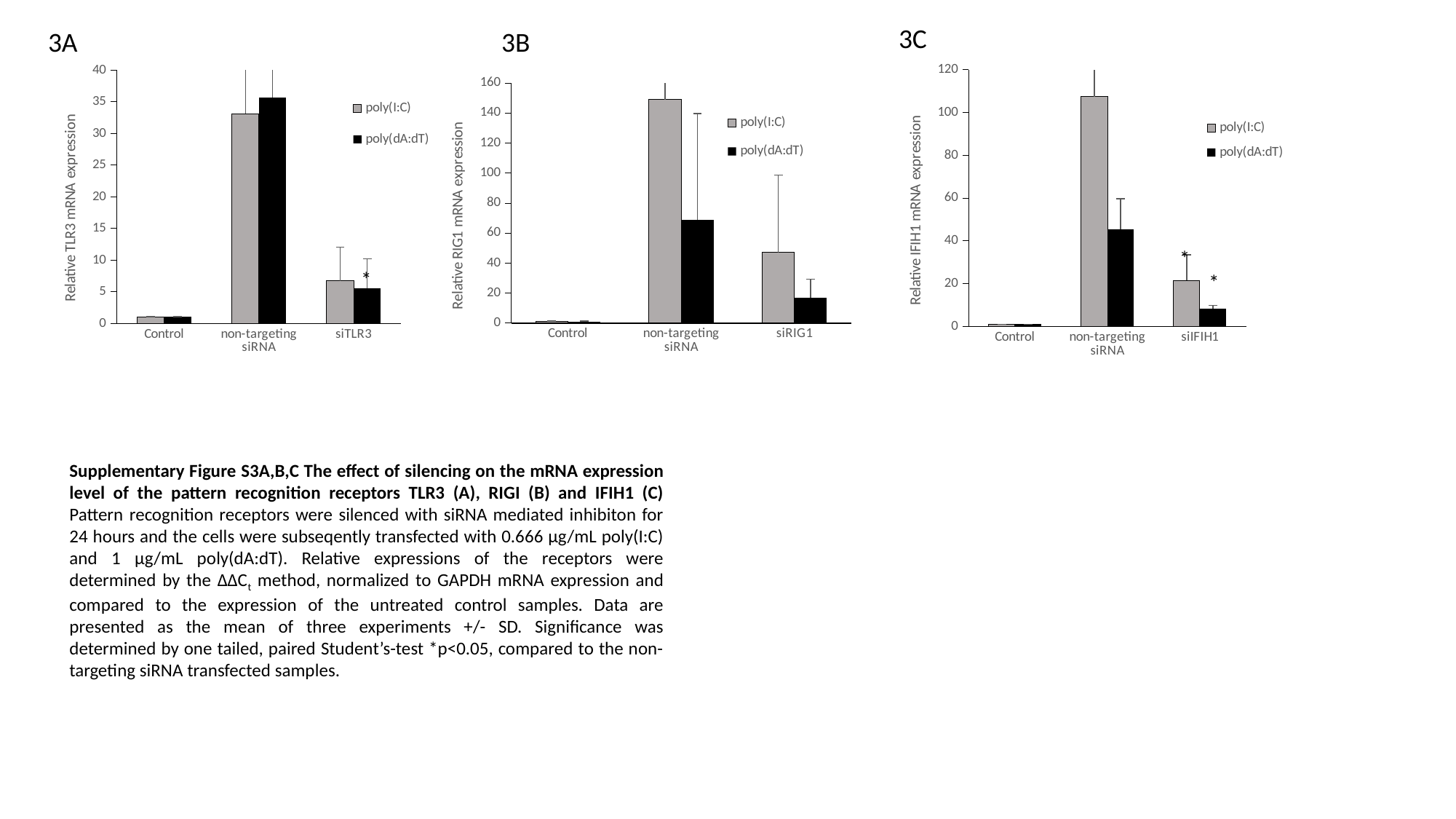

3C
3A
3B
### Chart
| Category | poly(I:C) | poly(dA:dT) |
|---|---|---|
| Control | 1.0351886675055633 | 1.0351886675055633 |
| non-targeting siRNA | 33.134983950761836 | 35.71415907038842 |
| siTLR3 | 6.716547698503469 | 5.52106526177683 |
### Chart
| Category | poly(I:C) | poly(dA:dT) |
|---|---|---|
| Control | 1.016355300271254 | 1.016355300271254 |
| non-targeting siRNA | 107.4949337460092 | 45.40875763238324 |
| siIFIH1 | 21.51214800261038 | 8.349752355125219 |
### Chart
| Category | poly(I:C) | poly(dA:dT) |
|---|---|---|
| Control | 1.1089889294526394 | 1.1089889294526394 |
| non-targeting siRNA | 148.9820063130332 | 68.99589225146167 |
| siRIG1 | 47.09740363374069 | 16.974296766054277 |*
*
*
Supplementary Figure S3A,B,C The effect of silencing on the mRNA expression level of the pattern recognition receptors TLR3 (A), RIGI (B) and IFIH1 (C) Pattern recognition receptors were silenced with siRNA mediated inhibiton for 24 hours and the cells were subseqently transfected with 0.666 μg/mL poly(I:C) and 1 μg/mL poly(dA:dT). Relative expressions of the receptors were determined by the ∆∆Ct method, normalized to GAPDH mRNA expression and compared to the expression of the untreated control samples. Data are presented as the mean of three experiments +/- SD. Significance was determined by one tailed, paired Student’s-test *p<0.05, compared to the non-targeting siRNA transfected samples.

## Slide 4
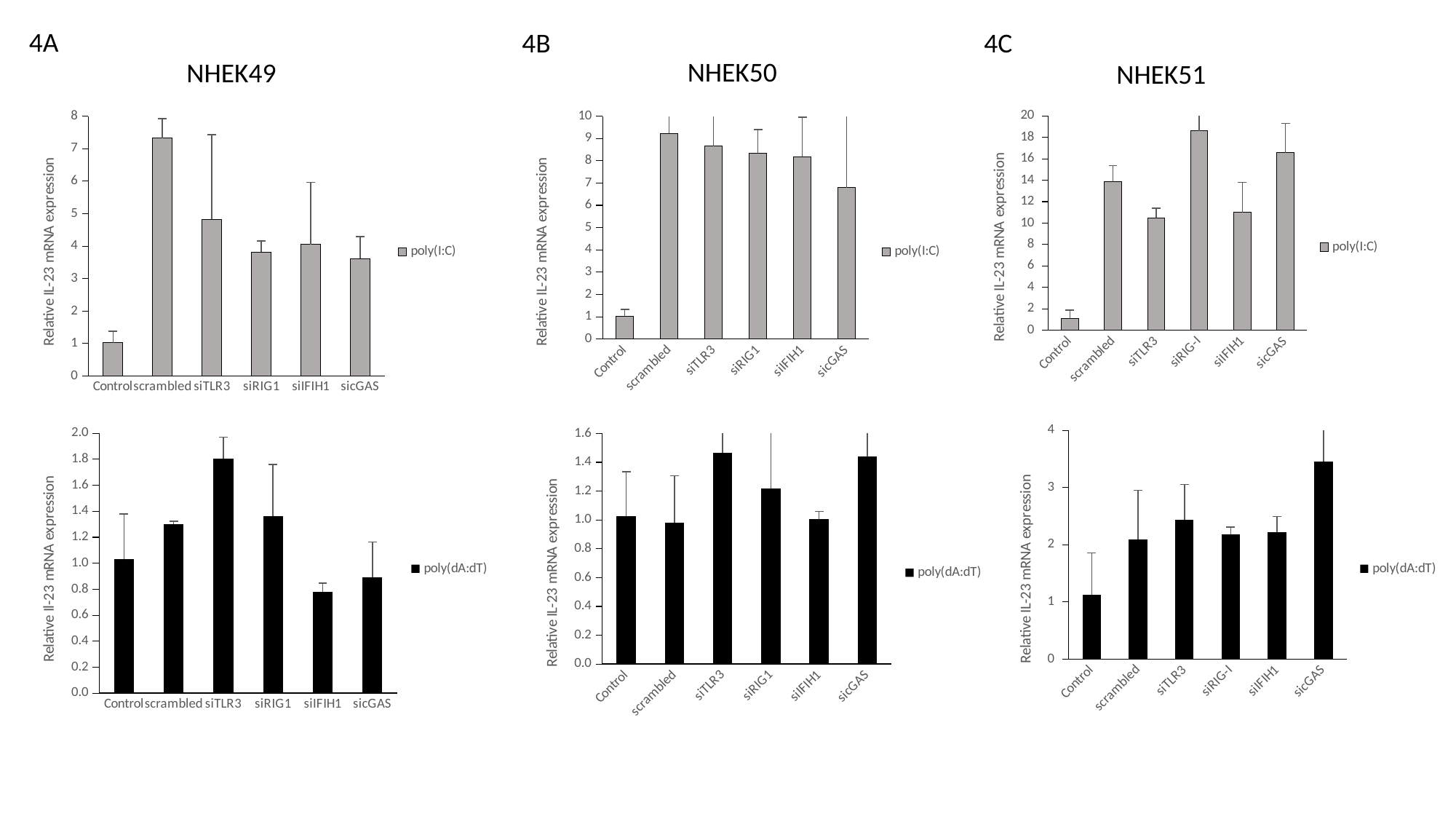

4A
4B
4C
NHEK50
NHEK49
NHEK51
### Chart
| Category | poly(I:C) |
|---|---|
| Control | 1.0298615598210625 |
| scrambled | 7.320479544892441 |
| siTLR3 | 4.825648823302468 |
| siRIG1 | 3.8160417890777207 |
| siIFIH1 | 4.048212899592004 |
| sicGAS | 3.6141001849335384 |
### Chart
| Category | poly(I:C) |
|---|---|
| Control | 1.0239268421020176 |
| scrambled | 9.217659031554387 |
| siTLR3 | 8.654422497001853 |
| siRIG1 | 8.344588566480047 |
| siIFIH1 | 8.176359891826595 |
| sicGAS | 6.788997378052553 |
### Chart
| Category | poly(I:C) |
|---|---|
| Control | 1.1258293412482998 |
| scrambled | 13.903879858759163 |
| siTLR3 | 10.494936543746936 |
| siRIG-I | 18.618672965325793 |
| siIFIH1 | 10.99154893059272 |
| sicGAS | 16.58611449674111 |
### Chart
| Category | poly(dA:dT) |
|---|---|
| Control | 1.1258293412482998 |
| scrambled | 2.0906244239915877 |
| siTLR3 | 2.430354490195743 |
| siRIG-I | 2.183021716102769 |
| siIFIH1 | 2.219860800301972 |
| sicGAS | 3.4510273544409937 |
### Chart
| Category | poly(dA:dT) |
|---|---|
| Control | 1.0298615598210625 |
| scrambled | 1.3010412764231887 |
| siTLR3 | 1.8023623831693634 |
| siRIG1 | 1.362346028827773 |
| siIFIH1 | 0.7781651533146018 |
| sicGAS | 0.8943235490289179 |
### Chart
| Category | poly(dA:dT) |
|---|---|
| Control | 1.0239268421020176 |
| scrambled | 0.9822918859189929 |
| siTLR3 | 1.4666261093284758 |
| siRIG1 | 1.2197311299826241 |
| siIFIH1 | 1.0058103950998203 |
| sicGAS | 1.4414841123571445 |

## Slide 5
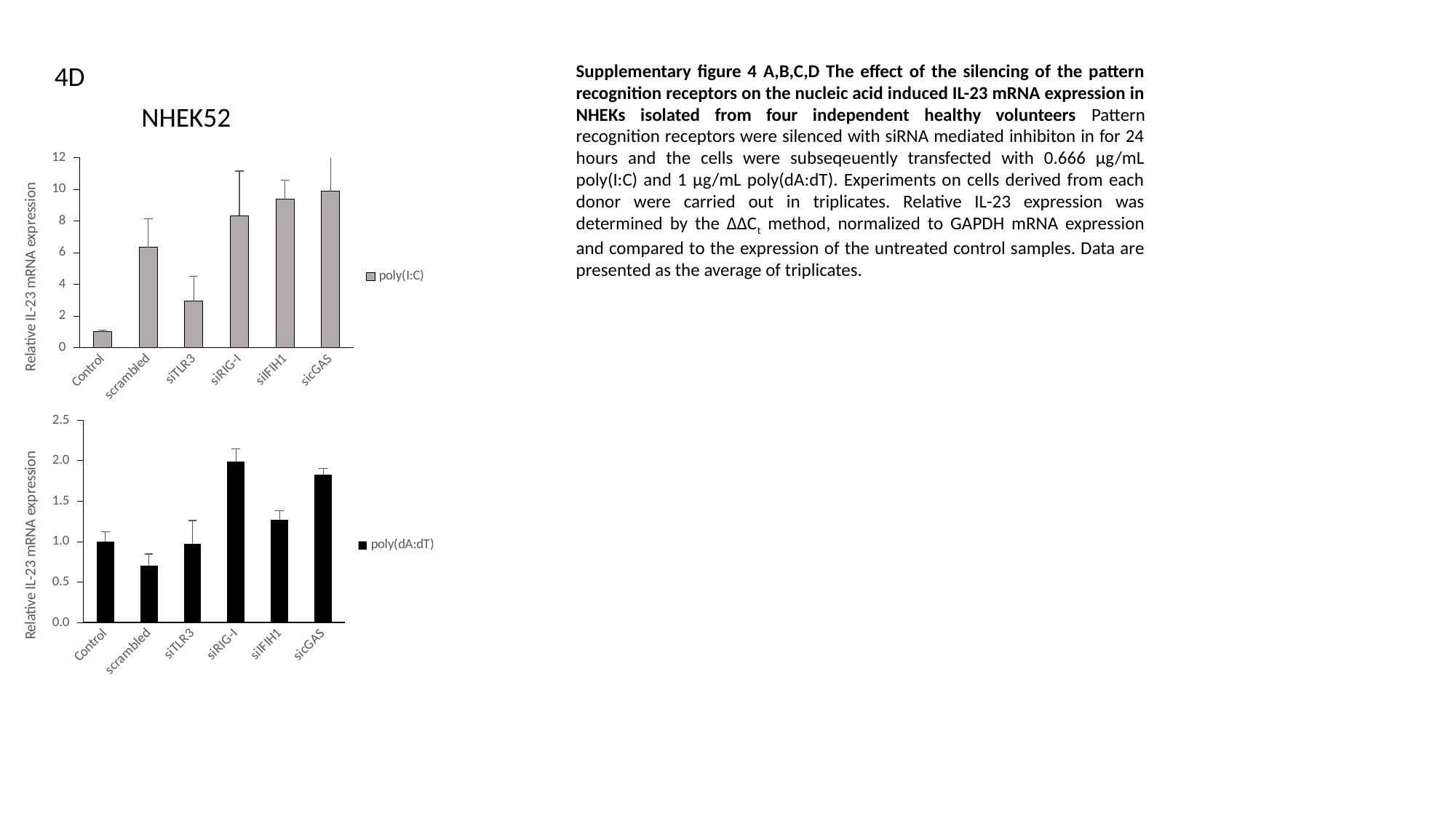

4D
Supplementary figure 4 A,B,C,D The effect of the silencing of the pattern recognition receptors on the nucleic acid induced IL-23 mRNA expression in NHEKs isolated from four independent healthy volunteers Pattern recognition receptors were silenced with siRNA mediated inhibiton in for 24 hours and the cells were subseqeuently transfected with 0.666 μg/mL poly(I:C) and 1 μg/mL poly(dA:dT). Experiments on cells derived from each donor were carried out in triplicates. Relative IL-23 expression was determined by the ∆∆Ct method, normalized to GAPDH mRNA expression and compared to the expression of the untreated control samples. Data are presented as the average of triplicates.
NHEK52
### Chart
| Category | poly(I:C) |
|---|---|
| Control | 1.003456907398978 |
| scrambled | 6.331249768277634 |
| siTLR3 | 2.9554744415488834 |
| siRIG-I | 8.342496782134813 |
| siIFIH1 | 9.397833519768836 |
| sicGAS | 9.917168349320832 |
### Chart
| Category | poly(dA:dT) |
|---|---|
| Control | 1.003456907398978 |
| scrambled | 0.703789620394067 |
| siTLR3 | 0.9736914934653499 |
| siRIG-I | 1.9926897537740544 |
| siIFIH1 | 1.2693475379877923 |
| sicGAS | 1.828262031490008 |

## Slide 6
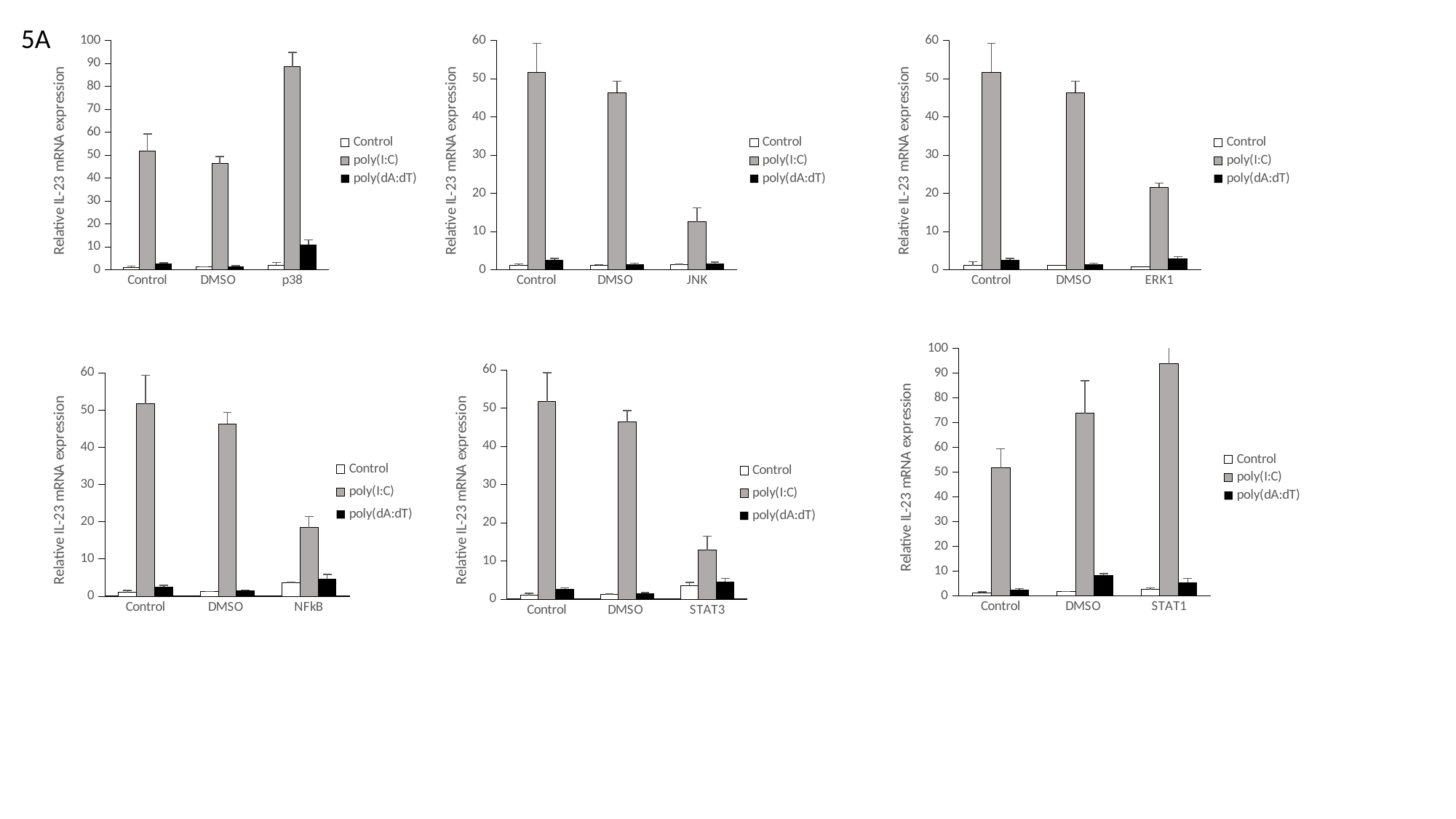

5A
### Chart
| Category | Control | poly(I:C) | poly(dA:dT) |
|---|---|---|---|
| Control | 1.0600938585392843 | 51.70257765256396 | 2.5948166252331486 |
| DMSO | 1.203273812383319 | 46.355544080438236 | 1.5217759795251475 |
| JNK | 1.4195525866143994 | 12.507429857659158 | 1.707012589564769 |
### Chart
| Category | Control | poly(I:C) | poly(dA:dT) |
|---|---|---|---|
| Control | 1.0600938585392843 | 51.70257765256396 | 2.5948166252331486 |
| DMSO | 1.203273812383319 | 46.355544080438236 | 1.5217759795251475 |
| ERK1 | 0.6986931367927622 | 21.562669391642984 | 2.9198199590191907 |
### Chart
| Category | Control | poly(I:C) | poly(dA:dT) |
|---|---|---|---|
| Control | 1.0600938585392843 | 51.70257765256396 | 2.5948166252331486 |
| DMSO | 1.203273812383319 | 46.355544080438236 | 1.5217759795251475 |
| p38 | 2.0248001512524847 | 88.59908257729174 | 11.096162062466798 |
### Chart
| Category | Control | poly(I:C) | poly(dA:dT) |
|---|---|---|---|
| Control | 1.0600938585392843 | 51.70257765256396 | 2.5948166252331486 |
| DMSO | 1.7128602830241757 | 73.78003839018163 | 8.229314645917436 |
| STAT1 | 2.553053574000077 | 93.71112865505503 | 5.503342091339141 |
### Chart
| Category | Control | poly(I:C) | poly(dA:dT) |
|---|---|---|---|
| Control | 1.0600938585392843 | 51.70257765256396 | 2.5948166252331486 |
| DMSO | 1.203273812383319 | 46.355544080438236 | 1.5217759795251475 |
| STAT3 | 3.6328152929697275 | 12.82399334967291 | 4.520814355751433 |
### Chart
| Category | Control | poly(I:C) | poly(dA:dT) |
|---|---|---|---|
| Control | 1.0600938585392843 | 51.70257765256396 | 2.5948166252331486 |
| DMSO | 1.203273812383319 | 46.355544080438236 | 1.5217759795251475 |
| NFkB | 3.6892739551073426 | 18.430049103333207 | 4.675099254265544 |

## Slide 7
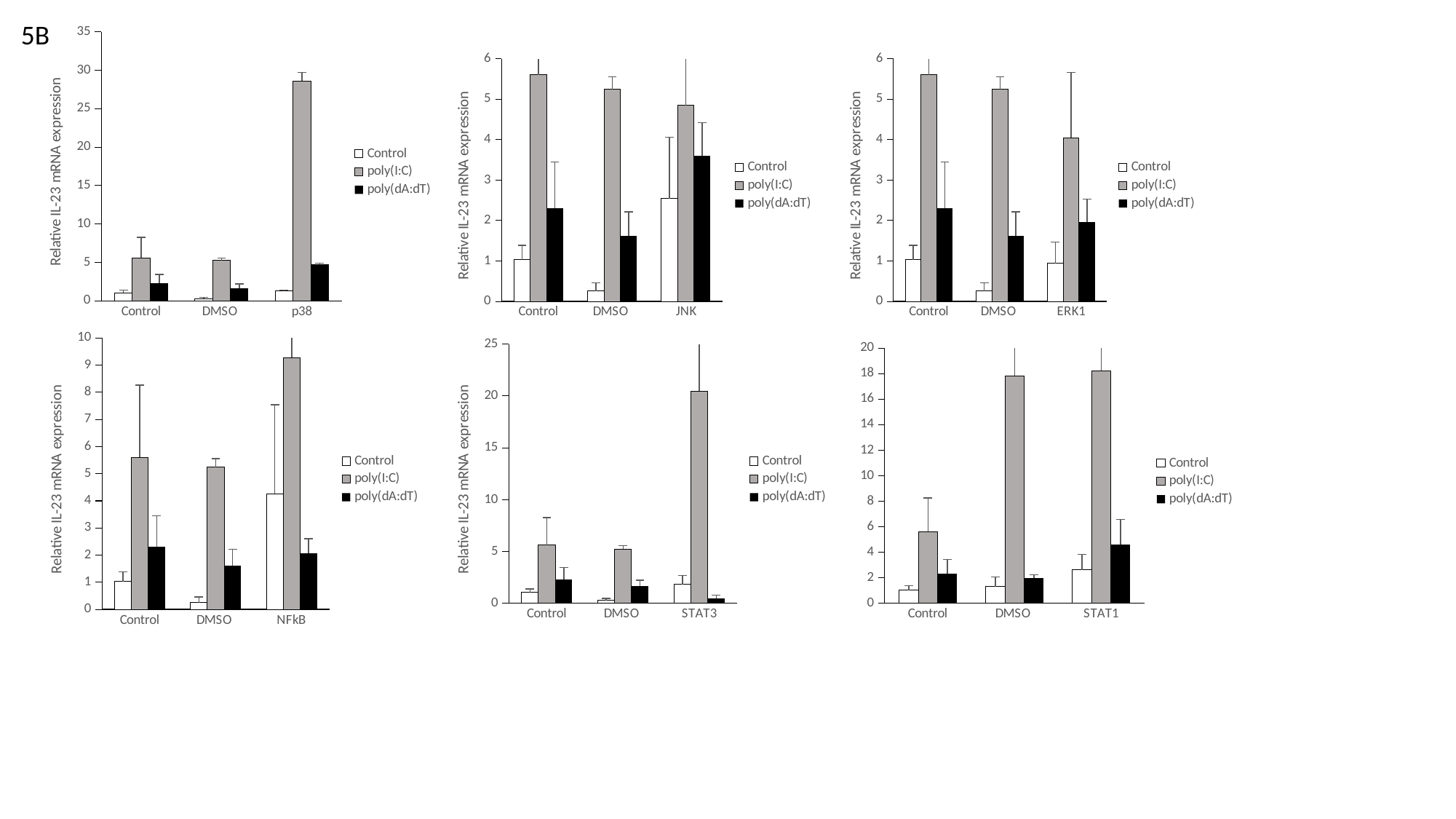

5B
### Chart
| Category | Control | poly(I:C) | poly(dA:dT) |
|---|---|---|---|
| Control | 1.0302079472530214 | 5.604412306804046 | 2.3100272723508963 |
| DMSO | 0.2579178155524556 | 5.237116090032796 | 1.622648359908724 |
| p38 | 1.3368071865088396 | 28.595851563277318 | 4.788428120722304 |
### Chart
| Category | Control | poly(I:C) | poly(dA:dT) |
|---|---|---|---|
| Control | 1.0302079472530214 | 5.604412306804046 | 2.3100272723508963 |
| DMSO | 0.2579178155524556 | 5.237116090032796 | 1.622648359908724 |
| JNK | 2.5375575585007217 | 4.841053552113139 | 3.6044025862151496 |
### Chart
| Category | Control | poly(I:C) | poly(dA:dT) |
|---|---|---|---|
| Control | 1.0302079472530214 | 5.604412306804046 | 2.3100272723508963 |
| DMSO | 0.2579178155524556 | 5.237116090032796 | 1.622648359908724 |
| ERK1 | 0.9440238434965851 | 4.040628755637238 | 1.9646440892522172 |
### Chart
| Category | Control | poly(I:C) | poly(dA:dT) |
|---|---|---|---|
| Control | 1.0302079472530214 | 5.604412306804046 | 2.3100272723508963 |
| DMSO | 0.2579178155524556 | 5.237116090032796 | 1.622648359908724 |
| NFkB | 4.262393266035704 | 9.280217269771764 | 2.072339102186044 |
### Chart
| Category | Control | poly(I:C) | poly(dA:dT) |
|---|---|---|---|
| Control | 1.0302079472530214 | 5.604412306804046 | 2.3100272723508963 |
| DMSO | 0.2579178155524556 | 5.237116090032796 | 1.622648359908724 |
| STAT3 | 1.83335489997455 | 20.475857992880666 | 0.47665942683788287 |
### Chart
| Category | Control | poly(I:C) | poly(dA:dT) |
|---|---|---|---|
| Control | 1.0302079472530214 | 5.604412306804046 | 2.3100272723508963 |
| DMSO | 1.3192873228960542 | 17.821306550381443 | 1.9675931000939335 |
| STAT1 | 2.6189898796349347 | 18.222875007107483 | 4.603023258101676 |

## Slide 8
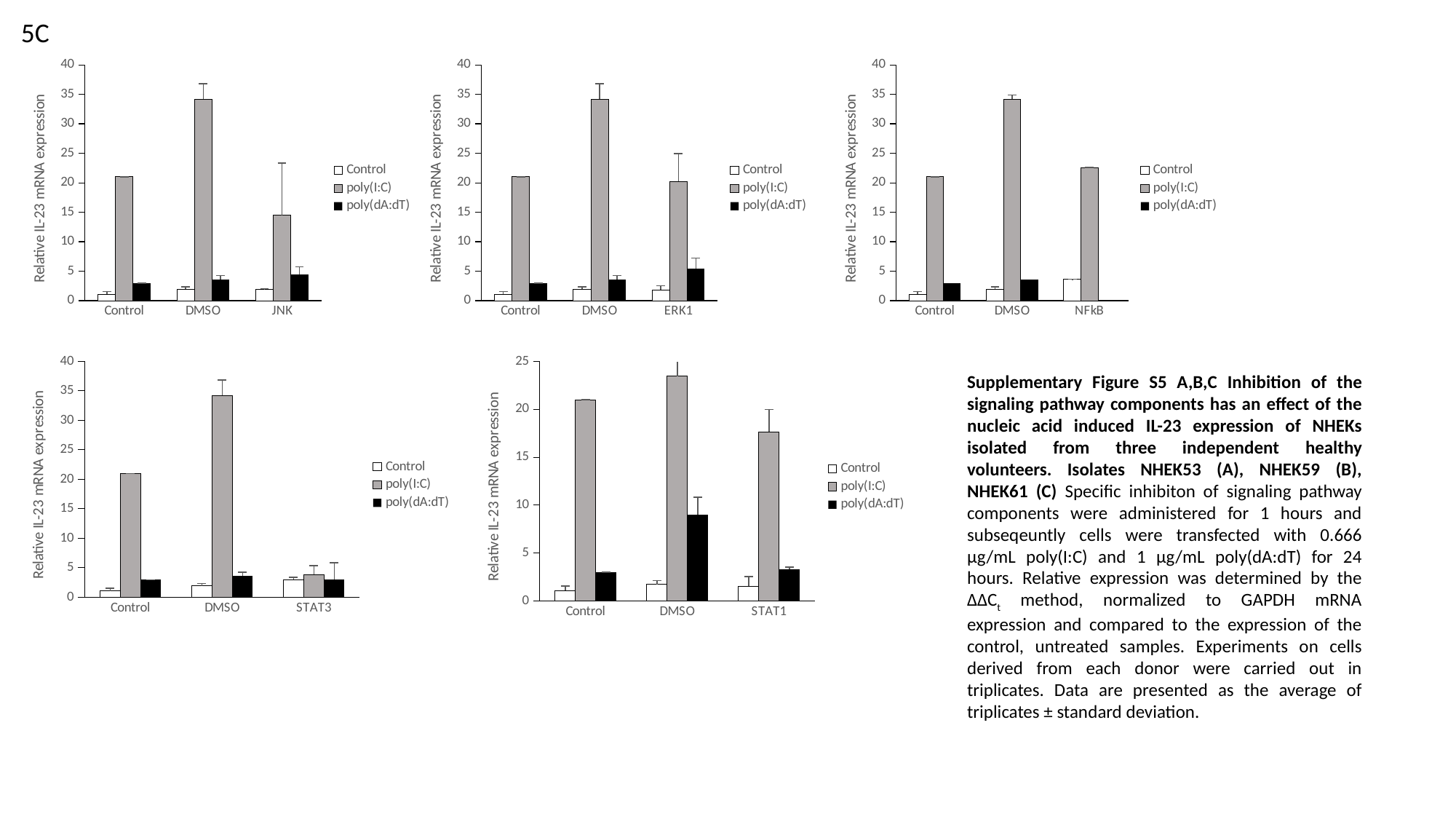

5C
### Chart
| Category | Control | poly(I:C) | poly(dA:dT) |
|---|---|---|---|
| Control | 1.0557058033884958 | 20.99609461727377 | 2.968635379108533 |
| DMSO | 1.954872495042717 | 34.18593418855351 | 3.5882258823566167 |
| JNK | 1.909934145220756 | 14.475977527784714 | 4.384051150134769 |
### Chart
| Category | Control | poly(I:C) | poly(dA:dT) |
|---|---|---|---|
| Control | 1.0557058033884958 | 20.99609461727377 | 2.968635379108533 |
| DMSO | 1.954872495042717 | 34.18593418855351 | 3.5882258823566167 |
| ERK1 | 1.8380667387459224 | 20.20826900616816 | 5.425866369791942 |
### Chart
| Category | Control | poly(I:C) | poly(dA:dT) |
|---|---|---|---|
| Control | 1.0557058033884958 | 20.99609461727377 | 2.968635379108533 |
| DMSO | 1.954872495042717 | 34.18593418855351 | 3.5882258823566167 |
| NFkB | 3.5819913479101517 | 22.564659466512005 | 0.0 |
### Chart
| Category | Control | poly(I:C) | poly(dA:dT) |
|---|---|---|---|
| Control | 1.0557058033884958 | 20.99609461727377 | 2.968635379108533 |
| DMSO | 1.7239049112271598 | 23.44285706519778 | 8.9949122945142 |
| STAT1 | 1.5145331234642259 | 17.661856237817346 | 3.266399258482724 |
### Chart
| Category | Control | poly(I:C) | poly(dA:dT) |
|---|---|---|---|
| Control | 1.0557058033884958 | 20.99609461727377 | 2.968635379108533 |
| DMSO | 1.954872495042717 | 34.18593418855351 | 3.5882258823566167 |
| STAT3 | 2.9684463564086556 | 3.868062749317165 | 2.9667812566144938 |Supplementary Figure S5 A,B,C Inhibition of the signaling pathway components has an effect of the nucleic acid induced IL-23 expression of NHEKs isolated from three independent healthy volunteers. Isolates NHEK53 (A), NHEK59 (B), NHEK61 (C) Specific inhibiton of signaling pathway components were administered for 1 hours and subseqeuntly cells were transfected with 0.666 μg/mL poly(I:C) and 1 μg/mL poly(dA:dT) for 24 hours. Relative expression was determined by the ∆∆Ct method, normalized to GAPDH mRNA expression and compared to the expression of the control, untreated samples. Experiments on cells derived from each donor were carried out in triplicates. Data are presented as the average of triplicates ± standard deviation.

## Slide 9
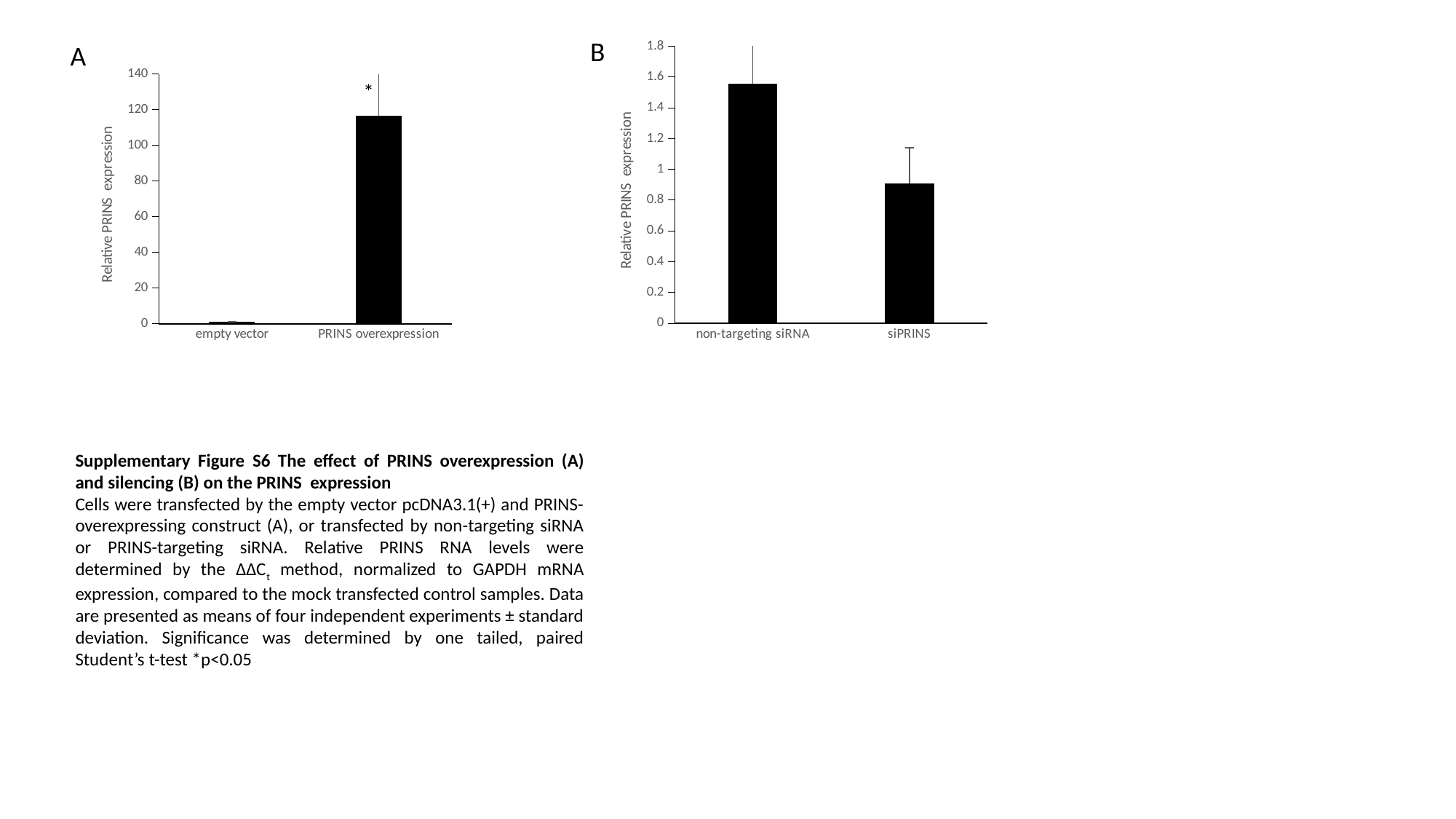

B
A
### Chart
| Category | |
|---|---|
| non-targeting siRNA | 1.5555429537568806 |
| siPRINS | 0.9068488966500552 |
### Chart
| Category | |
|---|---|
| empty vector | 1.0332507181274702 |
| PRINS overexpression | 116.5358588063522 |*
Supplementary Figure S6 The effect of PRINS overexpression (A) and silencing (B) on the PRINS expression
Cells were transfected by the empty vector pcDNA3.1(+) and PRINS-overexpressing construct (A), or transfected by non-targeting siRNA or PRINS-targeting siRNA. Relative PRINS RNA levels were determined by the ∆∆Ct method, normalized to GAPDH mRNA expression, compared to the mock transfected control samples. Data are presented as means of four independent experiments ± standard deviation. Significance was determined by one tailed, paired Student’s t-test *p<0.05

## Slide 10
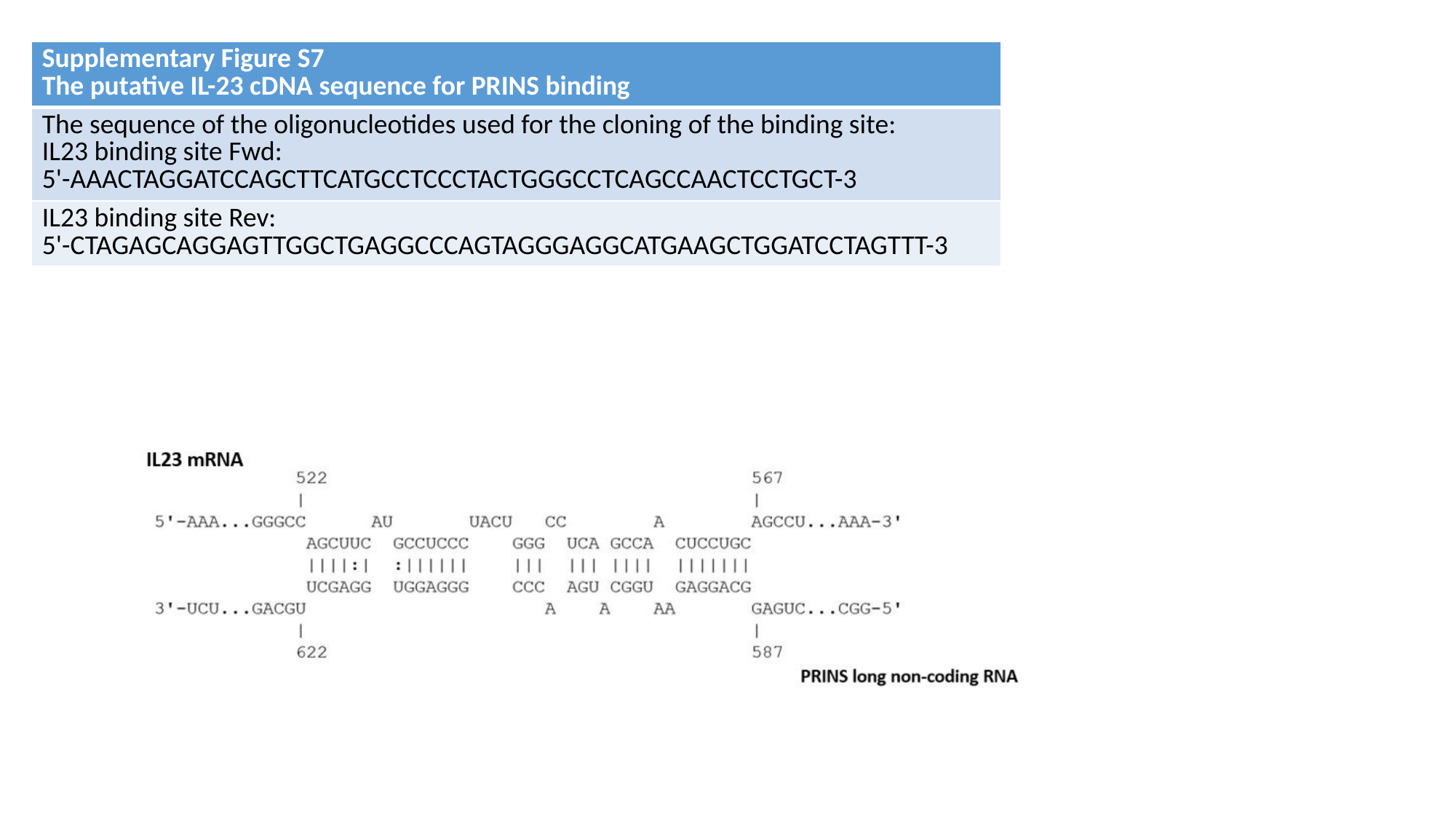

| Supplementary Figure S7 The putative IL-23 cDNA sequence for PRINS binding |
| --- |
| The sequence of the oligonucleotides used for the cloning of the binding site: IL23 binding site Fwd: 5'-AAACTAGGATCCAGCTTCATGCCTCCCTACTGGGCCTCAGCCAACTCCTGCT-3 |
| IL23 binding site Rev: 5'-CTAGAGCAGGAGTTGGCTGAGGCCCAGTAGGGAGGCATGAAGCTGGATCCTAGTTT-3 |
